# The ZIF-8 nanoplatform targeted delivery of IFI44 siRNA to suppress bladder cancer development via modulating the PI3K/AKT signaling pathway

**DOI:** 10.1186/s12951-026-04419-w

**Published:** 2026-04-30

**Authors:** Chao Zhu, Mengwei Liu, Xiaohua Liu, Sifan Zhang, Yun He, Meng Chen, Haoxuan Huang, Kuai Yu, Aiping Le

**Affiliations:** 1 Department of Transfusion Medicine, Key Laboratory of Jiangxi Province for Transfusion Medicine, The First Affiliated Hospital, Jiangxi Medical College, Nanchang University, Nanchang, 330006 Jiangxi China; 2 Department of Blood Transfusion, The Third Affiliated Hospital（the First Hosptial of Nanchang), Jiangxi Medical College, Nanchang University, Nanchang, China., Nanchang, 330006 Jiangxi China; 3 NHC Key Laboratory of Personalized Diagnosis and Treatment of Nasopharyngeal Carcinoma, Jiangxi Cancer Hospital, Nanchang, 330029 Jiangxi People’s Republic of China; 4 Department of Radiation Oncology, Jiangxi Cancer Hospital, Nanchang, 330029 Jiangxi People’s Republic of China; 5 Department of hematology, the First Affiliated Hospital of Nanchang University, Nanchang, 330006 China; 6 Department of Urology, The Third Affiliated Hospital（the First Hosptial of Nanchang), Jiangxi Medical College, Nanchang University, Nanchang, China, Nanchang, 330006 Jiangxi China

**Keywords:** IFI44, PI3K/AKT, ZIF-8, Targeted delivery, Bladder cancer

## Abstract

Malignant progression of bladder cancer (BC) is frequently caused by its complex molecular regulation, leading to unfavorable clinical outcomes. Our research indicates that interferon-induced protein 44 (IFI44) is significantly overexpressed in BC, thereby facilitating its malignant progression. Mechanistically, BC’s progression is impeded by the knockdown of IFI44, partially due to the inhibition of the PI3K/AKT signaling pathway. In addition, we constructed a Zeolitic Imidazolate Framework-8 (ZIF-8) nanoplatform, incorporating ZIF-8@siIFI44, ZIF-8@siIFI44@PEG and ZIF-8@siIFI44@PEG-RGD. Subsequent experiments demonstrated that ZIF-8@siIFI44 and ZIF-8@siIFI44@PEG possess anti-tumor property, while ZIF-8@siIFI44@PEG-RGD exhibited enhanced anti-tumor efficacy due to the inclusion of RGD targeting peptides which especially target tumors. Additionally, our study confirmed that the ZIF-8 nanoplatform enables targeted delivery of IFI44 siRNA, therefore preventing BC development partially by influencing the PI3K/AKT signaling pathway. Our findings identify IFI44 as an element that aids in the malignant development of BC, emphasizing its promise as a target for therapy.

**Graphical Abstract:**

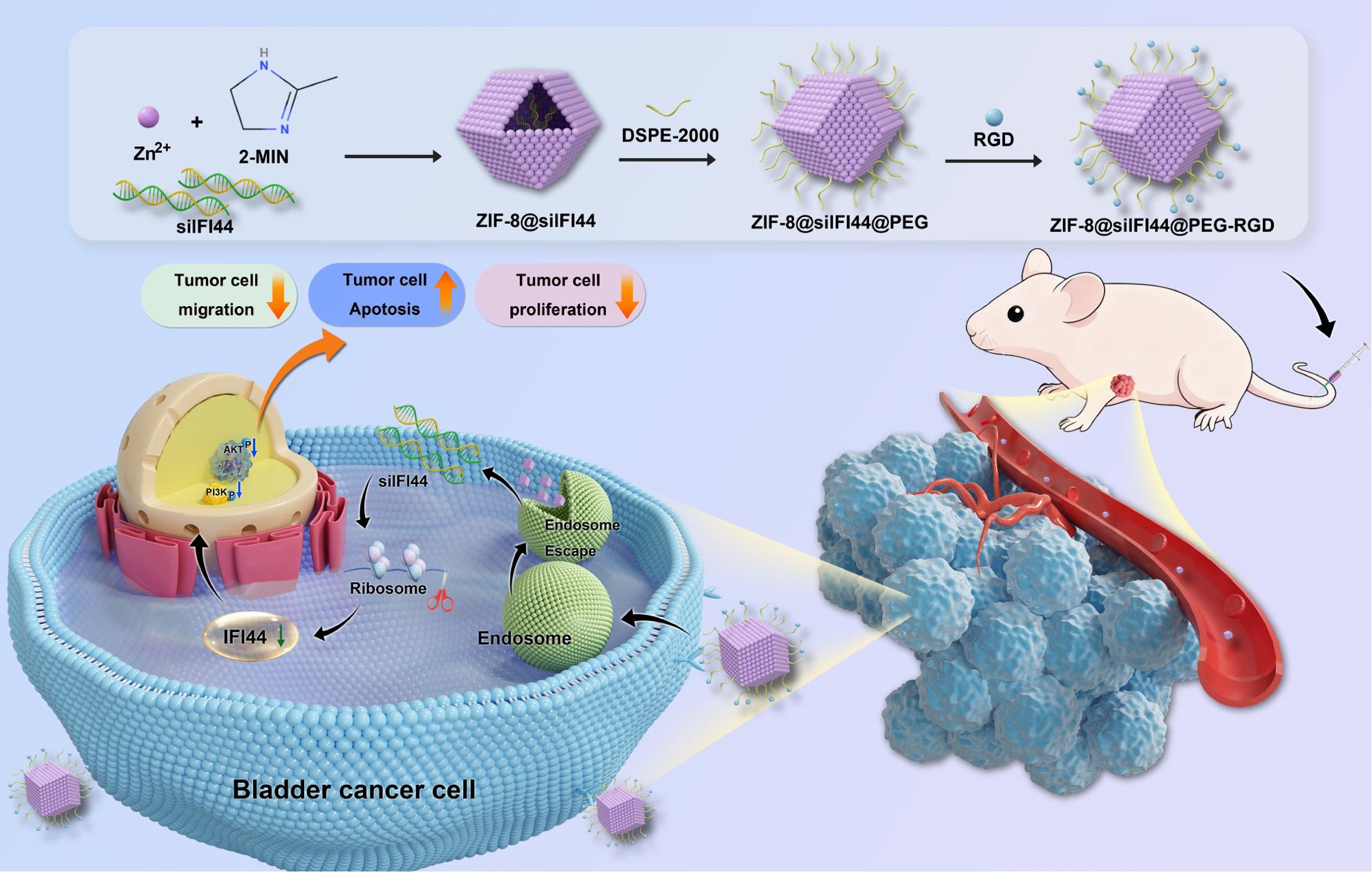

**Supplementary Information:**

The online version contains supplementary material available at 10.1186/s12951-026-04419-w.

## Introduction

In 2023, the International Agency for Research on Cancer (IARC) provided data showing [1], Bladder cancer (BC) ranks as the ninth most common malignant tumor globally. It is the sixth most prevalent malignant tumor in males and ranks second in frequency within the urinary system [1]. While mortality rates for BC have generally declined in most developed countries, they have increased in certain nations experiencing rapid economic transition [2]. Significant progress has been made in recent years regarding the treatment and prognosis of BC [3–5]. Notably, the combination of antibody-drug conjugates (ADCs) and immune checkpoint inhibitors (ICIs) has shown a synergistic effect in treating urogenital tumors, which has introduced novel therapeutic options for patients with BC [6]. The mortality and incidence rates of BC are affected by a variety of factors, including smoking [7, 8], occupational exposure [9, 10], and gender disparities [11]. Therefore, a comprehensive understanding of the molecular characteristics linked to tumorigenesis, along with the identification of novel prognostic biomarkers, is essential for improving clinical outcomes in patients with BC.

The interferon-inducible protein family includes Interferon-inducible protein 44 (IFI44) [12]. Investigations have found that IFI44 expression is higher in interstitial lung diseases related to systemic sclerosis and is closely related to disease progression [13]. Furthermore, the gene regulation of IFI44 varies notably in endothelial progenitor cells when exposed to monomeric versus pentameric C-reactive proteins, underscoring its role in immune regulation [14]. Recently, the involvement and mechanisms of IFI44 in tumorigenesis have garnered considerable attention. IFI44 has been shown to possess anti-cancer properties. Research indicates that the resistance of non-small cell lung cancer (NSCLC) cells to gefitinib is impacted by the long non-coding RNA LINC01116 via modulating IFI44 expression [15]. In an analysis of patients with NSCLC who underwent surgery, IFI44 was incorporated into a genetic model designed to predict recurrence-free survival [16]. Additionally, research findings reveal that the overexpression of LINCGAS5 markedly boosts the levels of IFI44 and STAT1, thereby inhibiting the proliferation and metastasis of papillary thyroid carcinoma [17]. However, in head and neck squamous cell carcinoma (HNSCC), IFI44 exhibits pro-cancer properties. For instance, in HNSCC, ACSL4 enhances interferon signaling, leading to increased expression of IFI44 and IFI44L, which in turn promotes HNSCC cell proliferation and invasiveness [18]. Further investigations need to target the detailed mechanisms of IFI44 across different tumor types to generate novel insights and strategies for cancer diagnosis and treatment. Our research endeavors to elucidate the role and clinical implications of IFI44 in the context of BC.

Numerous biological processes and pathologies are significantly influenced by the PI3K/AKT signaling pathway, with a notable impact on oncogenesis [19–22]. The association between PI3K/AKT signaling pathway and BC has attracted considerable scholarly interest in recent studies. Molecular profiling of BC has identified the PI3K/AKT/mTOR pathway could be a target for therapy in approximately 42% of cases [23]. Research indicates that the loss of chromosome 9q in non-muscle-invasive BC leads to enhanced PI3K/AKT/mTOR signaling [24]. Studies have shown that G3BP1 and SLU7 downregulate MHC-I expression through activation of this pathway, thereby augmenting the immune escape capacity of BC cells [25]. In the context of therapeutic applications, agents that block the PI3K/AKT signaling pathway have presented significant therapeutic potential [26, 27]. In summary, BC’s progression, pathogenesis and treatment heavily rely on the PI3K/AKT signaling pathway. Nonetheless, the molecular process through which IFI44 affects the PI3K/AKT signaling pathway is still unknown. A comprehensive study on whether the specific role of IFI44 in BC depends on the PI3K/AKT signaling pathway can reveal new targets and methods for accurate treatment of BC.

Within the metal-organic frameworks (MOFs) family, Zeolitic Imidazolate Framework-8 (ZIF-8) is a distinguished example, noted for its distinctive structural and functional attributes [28, 29]. Comprising zinc ions and imidazole ligands, ZIF-8 forms a porous framework characterized by a high specific surface area and remarkable chemical stability, this structural configuration endows ZIF-8 with significant potential for diverse applications, including gas adsorption, catalysis, drug delivery, and related domains [30, 31]. ZIF-8 serves as an effective support material for the incorporation of other metal nanoparticles to enhance catalytic activity [32]. In the biomedical domain, ZIF-8 has exhibited significant potential, particularly in oncological applications. For example, ZIF-8 can be employed to create light-responsive systems for precise drug delivery and release, thereby enhancing therapeutic outcomes [33]. ZIF-8-based nanomaterials have demonstrated substantial efficacy and potential when integrated with various therapeutic strategies, positioning themselves as a promising nanotechnology platform in cancer treatment [34]. Furthermore, ZIF-8 has been applied in the development of antibacterial biomaterials, which exhibit superior performance in wound healing and tissue regeneration [35, 36]. In conclusion, through comprehensive structural and functional analyses, its potential for practical applications can be further expanded, offering innovative approaches and methodologies for the development and application of novel materials [37]. In this article, we focus on the therapeutic effect of nano material ZIF-8 wrapped with siIFI44 in BC, utilizing biomaterials alongside targeted therapy creates an innovative treatment scheme for BC.

The research focuses on the impact of IFI44 on BC. Preliminary evidence shows that the absence of IFI44 can slow down the malignant development of BC cells partially by inactivating the PI3K/AKT signaling pathway. Our results indicate that ZIF-8@siIFI44 and ZIF-8@siIFI44@PEG effectively inhibit tumor growth, with ZIF-8@siIFI44@PEG-RGD exhibiting the most potent anti-tumor effect. These results could improve our comprehension of the molecular processes involved in BC progression and aid in creating new therapeutic approaches.

## Methods

### Cell lines and cell culture

Under standard laboratory conditions, T24, J82, and HEK-293T cell lines were maintained. RPMI 1640 medium was used to culture T24 and J82 cells, whereas DMEM was used for HEK-293T cells. The culture media contained an addition of 10% fetal bovine serum (FBS) (Cellmax, SA211.02, Beijing) and 1% penicillin/streptomycin to support cell growth and prevent bacterial contamination.

### Cell apoptosis analysis

For apoptosis experiments, we utilized the Annexin V-FITC/PI kit. On the first day, incubate 5 × 10^5^ cells in a six-well plate, then collect and resuspend them in buffer on the second day, followed by 10 min of FITC staining and 5 minutes of PI staining and finally analyze them using flow cytometry.

### Tissue specimens

We collected 16 pairs of fresh BC tissues and adjacent normal tissues with the help of urologists. Samples were preserved at -80 °C for later protein and mRNA level validation or immersed in 4% paraformaldehyde and enclosed in paraffin for (immunohistochemical staining) IHC. Then we searched 81 paraffin embedded BC tissues in the pathology department for IHC. The research was approved by the Ethics Committee of the First Affiliated Hospital of Nanchang University (Approval Number: (2025) CDYFYYLK (11–021)).

### siRNA, shRNA and cell transfection

The shRNA and siRNA primers were produced by General Bio (Anhui) Co., Ltd. (Anhui, China), the sequences can be found in Table S1. To create stable cell lines with reduced IFI44 expression, we used psPAX2, pMD2G, and the shIFI44#1/2 target plasmid for lentiviral packaging in HEK-293T cells. T24 and J82 cells were then infected using the viral supernatant that was produced. Subsequently, we identified stable cell lines that could downregulate IFI44 using puromycin and ultimately employed them for phenotype validation.

### Western blotting

The antibodies used included IFI44 (Proteintech, Cat# 68575-1-Ig), GAPDH (Huabio, Cat# ET1601-4), Bax (Servicebio, Cat# GB11690), bcl2 (Servicebio, Cat# GB154380), p-PI3K (ABclonal, Cat# AP0427), PI3K (Affinity, #AF6241), p-AKT (Proteintech, Cat# 80455-1-RR), AKT (Proteintech, Cat# 60203-2-Ig).

### Mouse xenograft model

The mouse xenograft tumor model is an experiment that establishes a pathological model by implanting human tumor cells into immunodeficient animals. In this experiment, we used the mouse xenograft tumor model. We injected 5 million cells from each group into the axilla of nude mice. The tumor’s volume was assessed every three days after it reached a particular size, using the calculation length × width²/2. At the point when the tumor’s longest diameter hit 15 mm, the tumor was extracted after the nude mice were euthanized and for mRNA and protein validation or for IHC detection. Hangzhou Ziyuan Laboratory Animal Technology Co., Ltd. supplied the female BALB/c nude mice. The animal research received approval from the Ethics Committee at the First Affiliated Hospital of Nanchang University (Ethics Number: CDYFY-IACUC-202403QR036).

### Synthesis of ZIF-8@siIFI44@PEG-RGD

The project included synthesizing ZIF-8 through the co-precipitation technique and loading of RNA onto ZIF-8 via the impregnation method, and the covalent coupling of RGD to the surface of ZIF-8.

### Characterization of ZIF-8@siIFI44@PEG-RGD

A Zetasizer Nano ZS90 (Malvern Instruments, UK) was used to measure the particle size and zeta potential of different ZIF-8 groups. Transmission electron microscopy (TEM, Japan) was used to characterize the morphology of different ZIF-8 groups. The encapsulation efficiency of siIFI44 was evaluated using ultraviolet-visible spectroscopy (UV-Vis), yielding a value of approximately 72.89%. Using the dialysis method, the release of siIFI44 from ZIF-8 was explored. Release efficiency of siIFI44 was assessed in both acidic (10 mM PBS solution, pH 6) and neutral (10 mM PBS solution, pH 7.4) environments under conditions of 37 °C and agitation at 200 rpm.

### Cellular uptake

In a confocal dish, BC cells were seeded and left to adhere permitted to adhere before ZIF-8, ZIF-8@siIFI44, ZIF-8@siIFI44@PEG and ZIF-8@siIFI44@PEG-RGD treatment for a duration of four hours. Subsequently, the BC cells underwent a one-hour incubation with Lyso-Tracker Green (Beyotime, China) to facilitate lysosomal staining. Nuclear staining was then conducted using Hoechst 33342 for ten minutes. Imaging was carried out with the help of a confocal laser scanning microscope (ZEISS, Germany).

### Pharmacokinetic assay and biodistribution of ZIF-8@siIFI44@PEG-RGD in mice

A random allocation process was used to divide healthy nude mice into two groups, with three mice in each group and each group receiving either naked siIFI44 or ZIF-8@siIFI44@PEG-RGD via tail vein injection at 1 nmol siRNA per mouse. Blood samples were then collected from the orbital sinus for additional experiments. The pharmacokinetics were assessed by quantifying the fluorescence intensity of CY5-labeled siIFI44 using an ELISA reader. In the biodistribution study, BALB/c nude mice bearing subcutaneous tumors were similarly divided into three groups, receiving an intravenous injection of either naked siIFI44, ZIF-8@siIFI44 or ZIF-8@siIFI44@PEG-RGD. The fluorescence intensity of CY5-siIFI44 in major organs and tumors was measured 24 h post-injection using the Milabs B.V. small animal CT/live imaging all-in-one machine.

### Anti-tumor efficacy and toxicity of ZIF-8@siIFI44@PEG-RGD

Initially, mice were administered axillary injections of 5 million T24 cells. On the ninth day following the injection, the mice were assigned randomly to five different groups. Commencing on the tenth day, each group received injections of ZIF-8, ZIF-8@siNC, ZIF-8@siIFI44, ZIF-8@siIFI44@PEG or ZIF-8@siIFI44@PEG-RGD every three days, for a total of six injections. On the 28th day, the tumors were excised from the euthanized mice for subsequent mRNA, western blotting and IHC experiments. Moreover, hematoxylin and eosin (HE) staining was performed on the heart, liver, spleen, lungs, and kidneys of the treated mice. To further evaluate potential toxicity, we collaborated with Servicebio (Wuhan, China) to assess the serum concentrations of creatinine (CREA), aspartate aminotransferase (AST), alanine aminotransferase (ALT) and urea, thereby determining the safety profile of the various ZIF-8 treatments in mice.

### Statistical analysis

Statistical analyses were conducted utilizing GraphPad Prism 9.0 and R. The data are displayed as the mean ± standard deviation, derived from three separate trials. The differences between two groups were assessed with a t-test, while one-way or two-way ANOVA was used for comparing multiple groups. A P-value threshold of under 0.05 was used to determine statistical significance.

## Results

### IFI44 is significantly higher in BC patients and BC cells

**Fig. 1:**
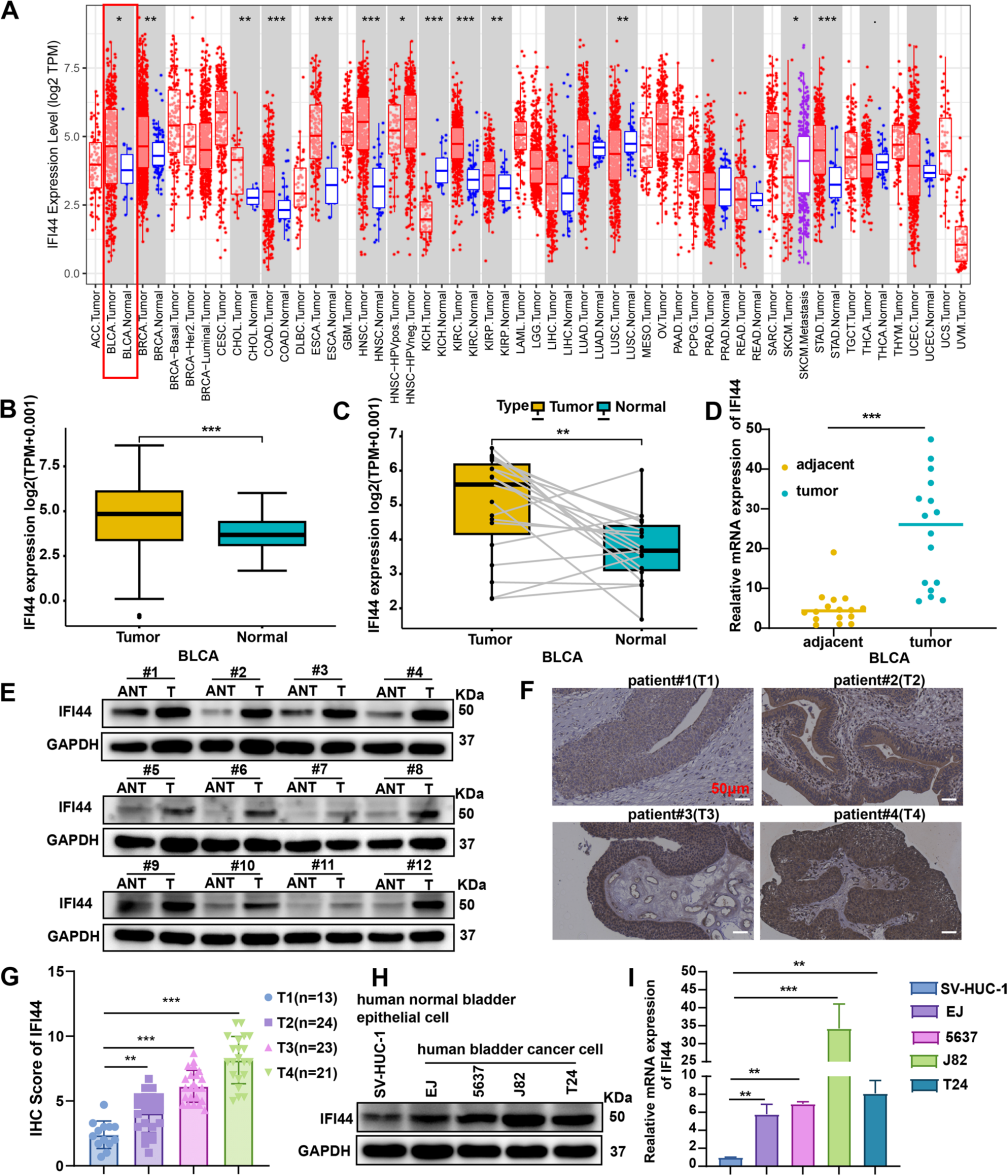
IFI44 is significantly higher in BC patients and BC cells. **(A)** The TCGA database was utilized to examine IFI44 gene expression across various tissue types. **(B)** Within BC tissues, IFI44 expression was notably elevated compared to normal tissues. **(C)** The TCGA dataset was employed to evaluate IFI44 gene expression in BC and adjacent normal tissues. **(D)** Quantitative analysis of IFI44 mRNA expression was conducted on sixteen pairs of BC tissues and their adjacent normal tissues. **(E)** Western blotting was performed on 12 pairs of BC and adjacent normal tissues to assess IFI44 protein levels. **(F)** Representative images of IFI44 immunohistochemical (IHC) staining for four BC tissues were provided, with a scale bar of 50μm. **(G)** Immunohistochemistry (IHC) scoring was employed to assess IFI44 expression in tumor tissues across these stages. **(H) **Western blotting was utilized to detect differences in IFI44 protein levels between normal bladder epithelial cells and BC cells.**(I)** RT-qPCR was employed to assess the differences in IFI44 RNA levels between normal bladder epithelial cells and BC cells. Data were presented as means ± SD. *P<0.05; **P<0.01; ***P<0.001. Experiments were repeated at least three times.

The expression levels of IFI44 were analyzed in different tumor types and their corresponding normal tissues using the pan-cancer RNA-seq datasets from the cancer genome atlas. According to the results, BC tissue exhibited a much higher expression of IFI44 than normal bladder epithelium (Fig. 1A). Moreover, the TCGA dataset also confirmed that the expression of IFI44 was elevated in tumor tissues, whether in unmatched BC patients or matched BC patients (Fig. 1B, C). To further validate the clinical importance of IFI44 expression in BC, we analyzed the mRNA levels of IFI44 in 16 fresh BC tissue pairs using RT-qPCR. A pronounced increase in IFI44 mRNA expression was observed in BC tissues (Fig. 1D). Furthermore, western blotting confirmed that IFI44 protein expression was significantly elevated in BC tissues compared to nearby normal tissues (Fig. 1E). Tumor staging in bladder cancer is primarily determined by the depth of tumor invasion into the bladder wall. In this study, we classified bladder cancer patients into stages T1, T2, T3, and T4, and assessed the expression levels of IFI44 in tumor tissues across these stages. As depicted in Fig. 1F, Patient 1 is classified as stage T1, Patient 2 as stage T2, Patient 3 as stage T3, and Patient 4 as stage T4. Immunohistochemical experiments have shown that elevated IFI44 expression was also related to the progression of tumor stages in patients, with higher expression of IFI44 leading to higher tumor staging (Fig. 1F). The quantitative analysis further illustrated that IFI44 expression levels increase concomitantly with advancing tumor stages (Fig. 1G). All above indicate that IFI44 is higher in BC tissue. Finally, we detected the IFI44 expression in human BC cells (EJ, 5637, J82, T24) and human normal bladder epithelial cells (SV-HUC-1). Western blot and RT-qPCR proved that IFI44 expression was higher in human BC cells than in human normal bladder epithelial cells (Fig. 1H, I). Consequently, we deduce that higher IFI44 levels are connected to unfavorable prognosis and might predict outcomes in BC.

### IFI44 facilitates the malignant advancement of BC cells in vitro and in vivo

**Fig. 2:**
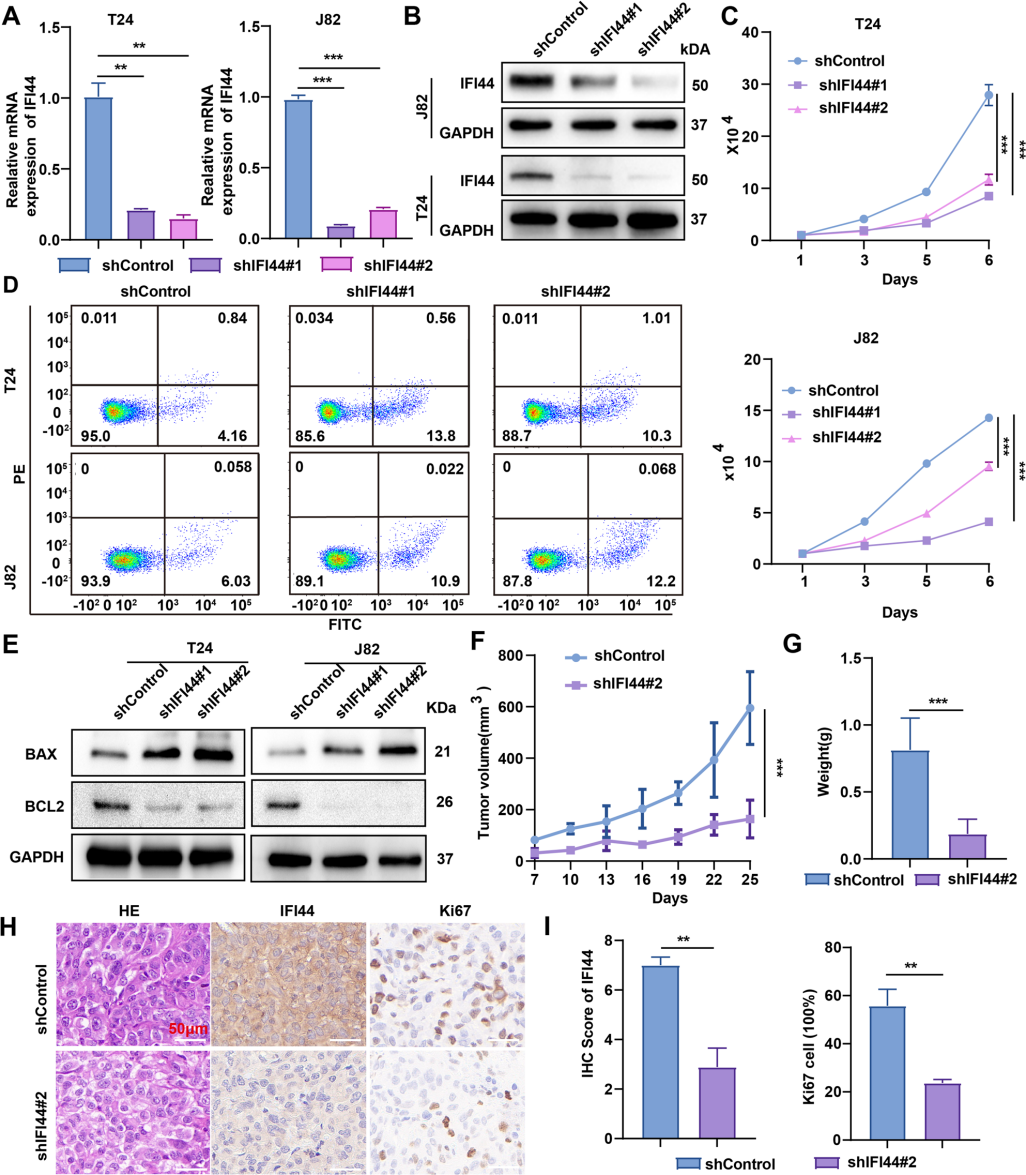
IFI44 facilitates the malignant advancement of BC cells in vitro and in vivo. **(A)** Assessment the knockdown of IFI44 efficiency at the RNA level in BC cells. **(B)** Evaluation of the knockdown of IFI44 efficiency at the protein level in BC cells. **(C)** Cell proliferation assays demonstrated the effect of the knockdown of IFI44 on BC cell proliferation. **(D)** Assessment of the apoptotic effects in the knockdown of IFI44 in T24 and J82 cells. **(E)** Analysis of expression levels of apoptosis-related proteins in T24 and J82 cells following the knockdown of IFI44. **(F)** Longitudinal monitoring of tumor growth in vivo. **(G)** Measurement of the weight of excised tumors. **(H)** IHC analysis of the knockdown of IFI44 efficiency and Ki67 expression levels in tumor tissues. **(I)** Evaluation of IFI44 and Ki67 protein levels in nude mouse tumor tissues using the IHC scoring method. Data are presented as means ± standard deviation (SD). **P < 0.01; ***P < 0.001. Experiments were repeated at least three times.

Considering IFI44’s pathological involvement in BC, we decided to examine its biological role in BC cells. Because of higher expression of IFI44 in T24 and J82 cells, we effectively knockdown of IFI44 in BC cell (Fig. 2A, B). Subsequently, we assessed the impact of IFI44 on the proliferation of BC cells through cell viability tests. The findings showed that the silencing of IFI44 significantly hindered the growth of T24 and J82 cells (Fig. 2C). Our findings interestingly revealed that the lowered viability of BC cells caused by the knockdown of IFI44 was not only due to a decrease in proliferative ability but also due to the promotion of apoptosis. The apoptosis analysis confirmed that the downregulation of IFI44 facilitates apoptosis in BC cells (Fig. 2D and Figure S2A). Through a transwell assay, we examined the ability of IFI44 in the metastatic potential of BC cells and discovered that the knockdown of IFI44 decreases the migratory ability of T24 and J82 cells (Figure S2B, C). Finally, the consistent changes in apoptosis-related proteins Bax and bcl2 suggested that the silencing of IFI44 promoted apoptosis in BC cells (Fig. 2E). To examine IFI44’s role in vivo, we employed a mouse xenograft tumor assay in which both the control and experimental groups of nude mice were administered subcutaneous injections of approximately 5 million T24 cells. The control group received cells infected with shControl, whereas the experimental group was injected with cells infected with shIFI44#2. Tumor length and width were measured every three days throughout the study, permitting natural growth of the tumors until they reached a maximum diameter of 15 mm, at which point the mice were euthanized. The results revealed that the knockdown of IFI44 expression significantly curtailed tumor growth in BALB/c nude mice (Fig. 2F, G and Figure S2D, E). Next, we performed immunohistochemistry in nude mouse tumors and detected changes in IFI44 and Ki67. The research proved that in the shIFI44#2 group, IFI44 was knocked down significantly, and after knocking down of IFI44, the proliferation index Ki67 was also significantly lower than the shControl group (Fig. 2H, I). Overall, our results indicate that the downregulation of IFI44 exhibits an anti-tumor ability on BC cells.

### BC’s progression is impeded by the knockdown of IFI44, partially due to the inhibition of the PI3K/AKT signaling pathway

**Fig. 3:**
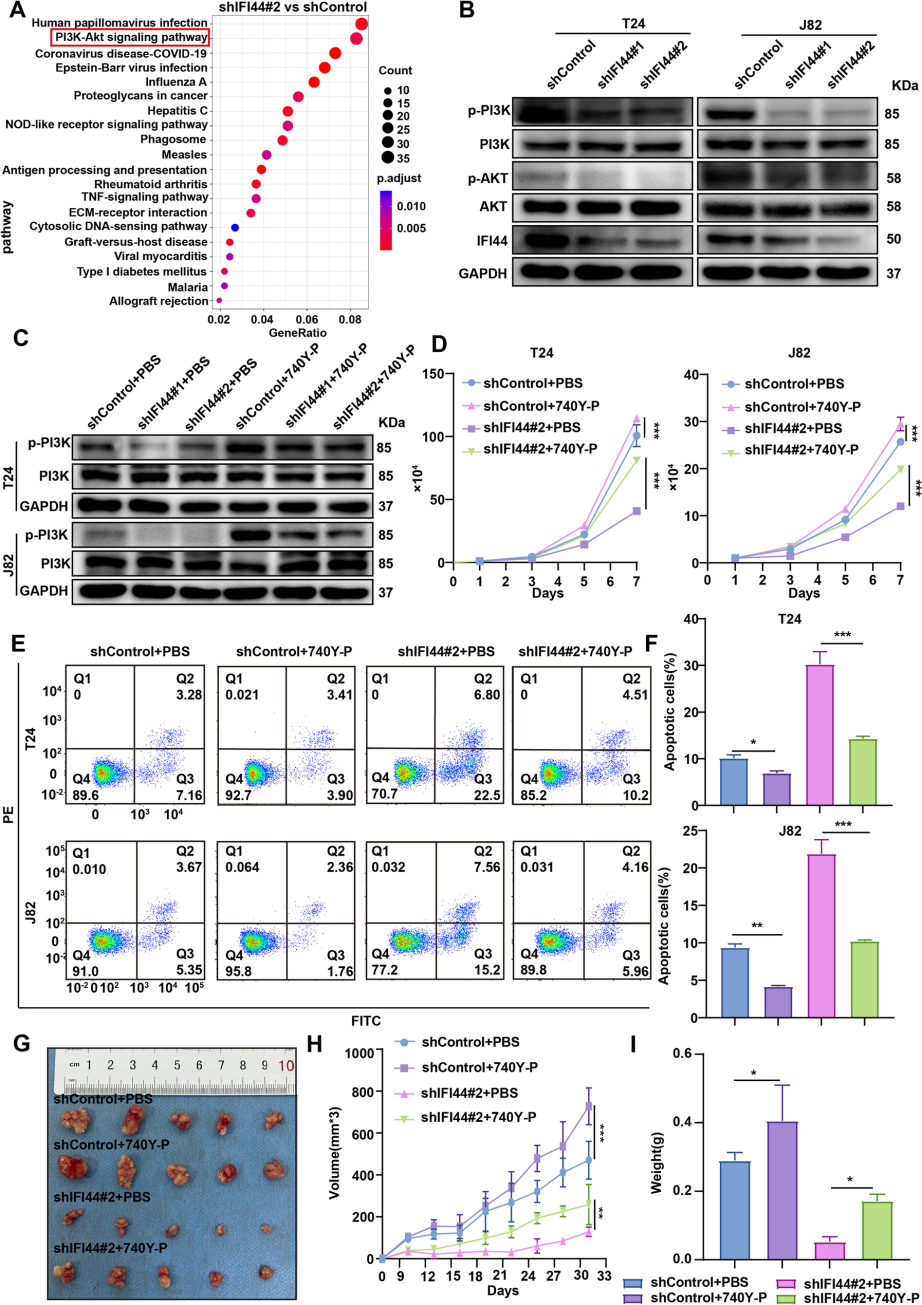
BC’s progression is impeded by the knockdown of IFI44, partially due to the inhibition of the PI3K/AKT signaling pathway. **(A)** Conducted KEGG enrichment analysis for differentially expressed genes in T24 cells with shControl and shIFI44#2. **(B)** Evaluated alterations in protein levels of total and phosphorylation levels of PI3K and AKT following the knockdown of IFI44 in BC cells using western blot. **(C)** Following the knockdown of IFI44 in BC cells, the PI3K agonist 740Y-P was administered to assess changes in total and phosphorylation of PI3K protein levels. **(D)** Cell counting assay was employed to assess the proliferative impact of 740Y-P on BC cells with the knockdown of IFI44. **(E)** Cell apoptosis assay was conducted to determine the apoptosis rate following the addition of 740Y-P to BC cells with the knockdown of IFI44. **(F)** Quantification of the apoptosis rate in BC cells with the knockdown of IFI44 following 740Y-P treatment. **(G)** Visual depiction of tumor samples. **(H)** Longitudinal assessment of tumor growth in vivo. **(I)** Measurement of tumor weight post-excision (*n* = 5). Data are expressed as means ± standard deviation (SD). *P < 0.05; **P < 0.01; ***P < 0.001. Experiments were repeated at least three times.

In order to understand the mechanism of IFI44 mediated malignant progression of BC, we performed RNA sequencing. Then we drew a volcano map (Figure S3A) and performed KEGG enrichment analysis, which identify that IFI44 may mediate's signaling pathway. The PI3K/AKT pathway was significantly enriched in T24 cells with the downregulation of IFI44 according to KEGG enrichment analysis (Fig. 3A). This pathway is intricately associated with the regulation of the cell cycle [38–40] and serves as a critical modulator of cellular metastasis [41–43], encompassing both invasion and migration processes. Consequently, it has emerged as a central focus in research on diseases such as cancer, due to its pivotal role in coordinating cell cycle progression and metastatic potential. Therefore, in this study, we focused on the role of PI3K/AKT signaling pathway in BC. Western blot analysis revealed that the lower IFI44 expression in T24 and J82 cells inhibits the PI3K/AKT signaling pathway, evidenced by reduced phosphorylation levels of PI3K and AKT, while total PI3K and AKT remained unchanged **(**Fig. 3B**)**. Hence, we deduced that IFI44 is involved in the malignant progression of BC cells partially by influencing the PI3K/AKT signaling pathway. To further demonstrate the reliability of this pathway, we chose the PI3K agonist 740Y-P for rescue experiments. Western blot proved that PI3K agonist 740Y-P can effectively activate the phosphorylation of the PI3K (Fig. 3C). Subsequently, we assessed the impact of 740Y-P on the proliferation of BC cells with the knockdown of IFI44 through cell viability assays and colony formation. The results showed that the addition of 740Y-P counteracts the inhibitory effect of the downregulation of IFI44 on BC cell proliferation (Fig. 3D and Figure S3B, C). In addition, the Apoptosis experiment indicated that the 740Y-P can mitigate the pro-apoptotic effects induced by the knockdown of IFI44 (Fig. 3E, F). Following that, we employed a mouse xenograft tumor model in conjunction with PI3K agonists (740Y-P) to investigate that BC’s progression is impeded by the knockdown of IFI44, partially due to the inhibition of the PI3K/AKT signaling pathway. Initially, both control and experimental groups of nude mice received subcutaneous injections of approximately 5 million T24 cells transfected with either shControl or shIFI44#2. Once the maximum tumor volume reached 100 mm³, the mice were subjected to intraperitoneal injections of either 740Y-P or PBS. Ultimately, when the maximum tumor diameter reached 15 mm, the mice were euthanized. Experiments with subcutaneous xenografts showed that the decrease in tumor formation due to the downregulation of IFI44 in T24 cells was notably counteracted by 740Y-P (Fig. 3G, H) and the use of 740Y-P did not affect the weight of mice (Figure S3D). The weight and volume of the dissected tumor all confirmed that 740Y-P can reverse the reduction of tumor caused by the knockdown of IFI44 in mice (Fig. 3I and Figure S3E). Overall, BC’s progression is impeded by the knockdown of IFI44, partially due to the inhibition of the PI3K/AKT signaling pathway.

### The Biological Characteristics of ZIF-8@siIFI44@PEG-RGD

**Fig. 4:**
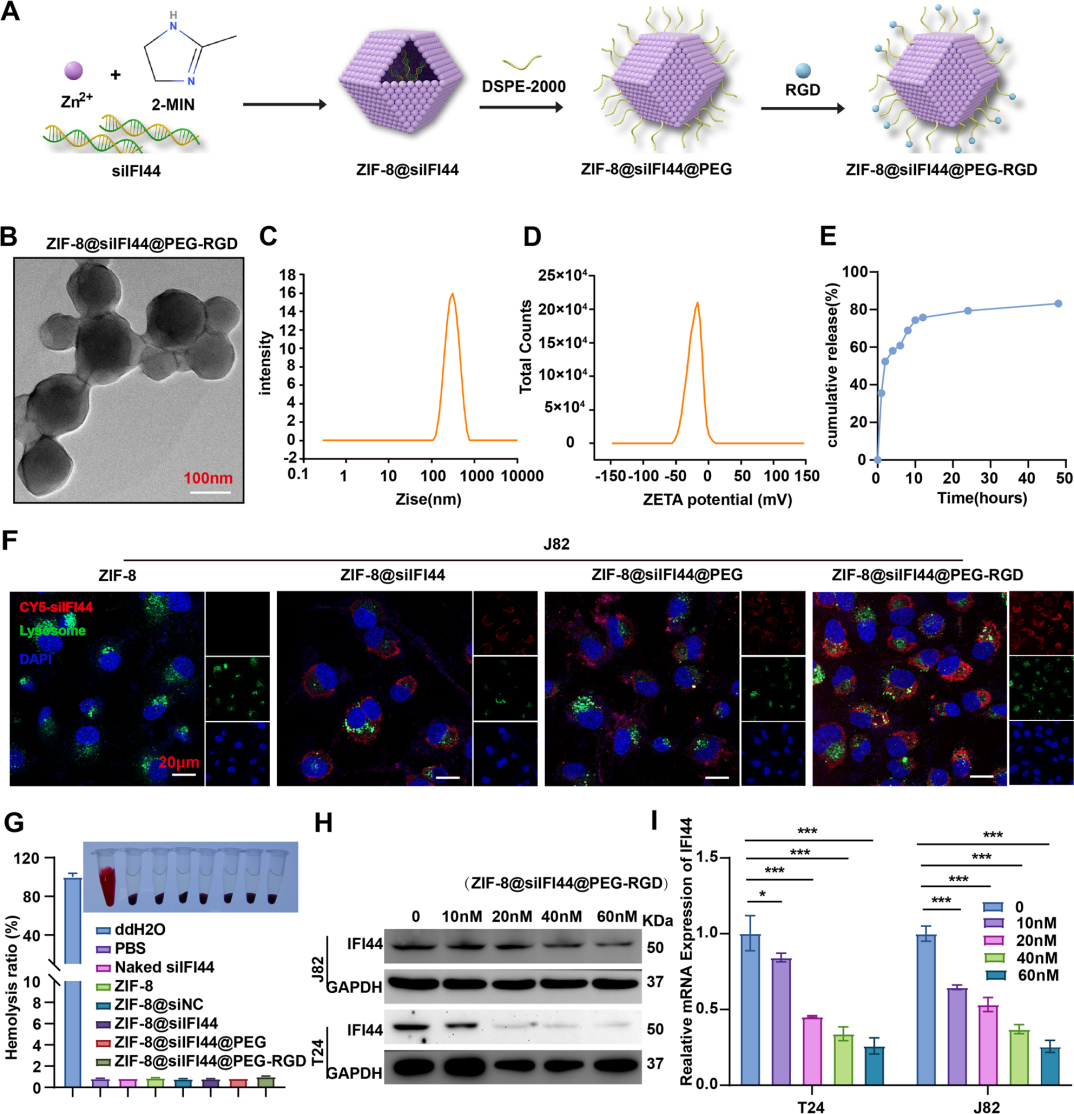
The Biological Characteristics of ZIF-8@siIFI44@PEG-RGD. **(A)** Illustration of the synthesis process for ZIF-8@siIFI44@PEG-RGD. **(B)** Transmission electron microscopy image of ZIF-8@siIFI44@PEG-RGD, with scale bars indicating 100 nm. **(C-D)** Dynamic light scattering analysis was employed to determine the particle sizes and zeta potentials of ZIF-8@siIFI44@PEG-RGD. **(E)** Cumulative release profile of siIFI44 in PH6.0 from ZIF-8@siIFI44@PEG-RGD at various time intervals. **(F)** Confocal laser scanning microscopy images of J82 cells following a 4-hour incubation with ZIF-8, ZIF-8@siIFI44, ZIF-8@siIFI44@PEG or ZIF-8@siIFI44@PEG-RGD. Nuclei were stained blue using Hoechst 33342, endosomes were visualized in green with Lysotracker, and siIFI44 was labeled with CY5, with scale bars set at 20 μm. **(G)** Hemolysis of erythrocytes was observed after a 2-hour incubation with PBS, deionized water or various ZIF-8. **(H)** The efficiency of the knockdown of IFI44 in BC cells was assessed via western blotting following treatment with varying concentrations of ZIF-8@siIFI44@PEG-RGD. **(I)** Quantitative RT-PCR was used to evaluate the knockdown efficiency of IFI44 in BC cells after treatment with different concentrations of ZIF-8@siIFI44@PEG-RGD. Data are expressed as means ± standard deviation (SD). *P < 0.05; ***P < 0.001. Experiments were repeated at least three times.

To confirm the anti-tumor activity of siIFI44 in vivo, we created different nanoparticles, including ZIF-8, ZIF-8@siNC, ZIF-8@siIFI44, ZIF-8@siIFI44@PEG and ZIF-8@siIFI44@PEG-RGD. Figure 4A has clearly described the synthesis process of ZIF-8@siIFI44@PEG-RGD. Our first step was to evaluate the physicochemical features of the different groups of nanomaterials. ZIF-8@siIFI44@PEG-RGD is identified as a rhombic dodecahedron through Transmission electron microscopy (TEM) analysis (Fig. 4B). The nanomaterials of other groups also have the same shape (Figure S4A). We choose Zetasizer Nano ZS90 to measure the particle size and potential of nanomaterials. The results indicated that the average diameter of ZIF-8@siIFI44@PEG-RGD is approximately 315.9 nm, with a potential of -22.7mV (Fig. 4C, D). Next, we also tested the average diameters and potentials of ZIF-8, ZIF-8@siNC, ZIF-8@siIFI44, ZIF-8@siIFI44@PEG, which average diameters were 177.4 nm, 198.4 nm, 241.8 nm, 229.7 nm respectively and the potentials were 31.9mV, -35.3mV, -31.9mV and -18.7 mV respectively (Figure S4B, C). By labeling siIFI44 with fluorescent dye CY5 (referred to as CY5-siIFI44), it was found that the encapsulation efficiency of siIFI44 was approximately 72.89%. We performed siRNA release curves in pH 6.0 PBS, which is a simulated tumor environment, and siRNA release curves in pH 7.4, which is a neutral environment. The dialysis test results showed that the cumulative release of siIFI44 under neutral conditions reached 51.51% within 48 h (Figure S4D), while under acidic conditions it reached 83.2% within 48 h (Fig. 4E). The results showed that the siIFI44 release of ZIF-8@siIFI44@PEG-RGD was successful. High uptake levels and effective lysosomal release were crucial for successful gene delivery and expression. After incubating ZIF-8, ZIF-8@siIFI44, ZIF-8@siIFI44@PEG and ZIF-8@siIFI44@PEG-RGD with BC cells for four hours, we found that most of the siIFI44 (marked by red fluorescence) managed to exit the endosomes (marked by green fluorescence) and move into the cytoplasm in ZIF-8@siIFI44, ZIF-8@siIFI44@PEG and ZIF-8@siIFI44@PEG-RGD. The endosomal escape of ZIF-8@siIFI44@PEG-RGD showed the most significant effect, conversely, the ZIF-8 showed very little red fluorescence (Fig. 4F and Figure S4E). Assessing the biocompatibility of materials requires considering their compatibility with blood. Significant hemolysis was observed in the positive control group through visual inspection, whereas the ZIF-8 group and the negative control groups did not show any detectable hemolysis. The hemolysis rate of all ZIF-8 groups was consistently found to be under 5% according to the quantitative analysis using an enzyme-linked immunosorbent assay (Fig. 4G). Ultimately, both western blot and RT-qPCR demonstrated that ZIF-8@siIFI44@PEG-RGD significantly decreased IFI44 expression in BC cells, nearly completely suppressing it at a siRNA concentration of 40 nM (Fig. 4H, I). The experimental findings indicate that ZIF-8@siIFI44@PEG-RGD was successfully created, showing high release efficiency, significant cellular uptake, favorable safety profile and strong knockdown efficiency.

### In vitro, ZIF-8@siIFI44@PEG-RGD hinders the malignant development of BC cells, partially due to the inhibition of the PI3K/AKT signaling pathway

**Fig. 5:**
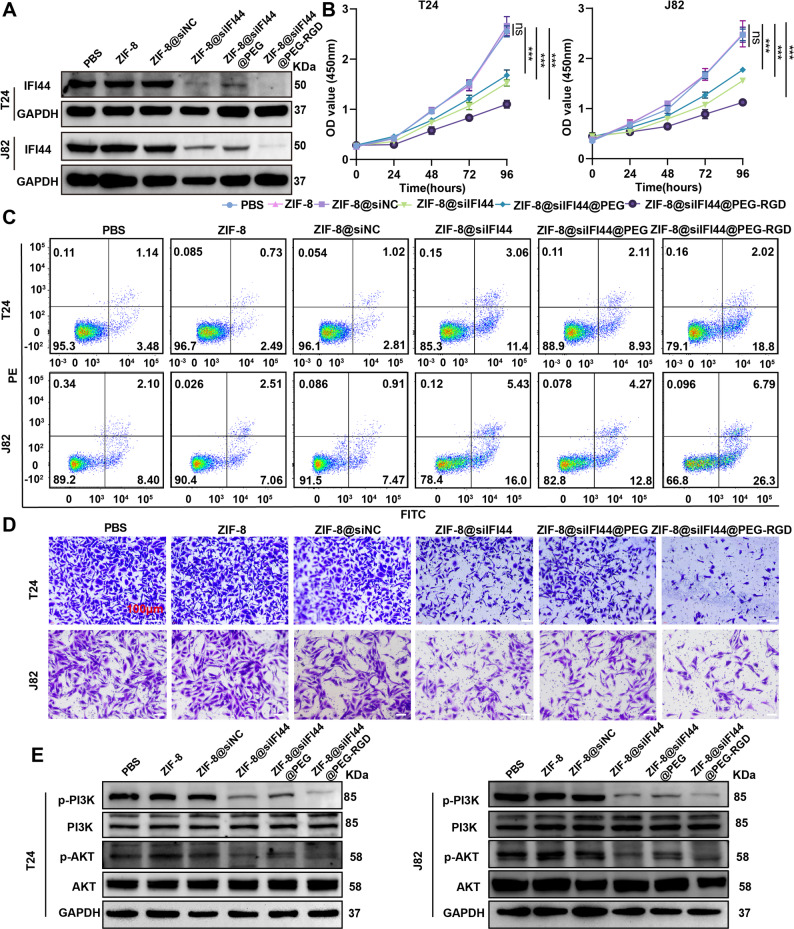
In vitro, ZIF-8@siIFI44@PEG-RGD hinders the malignant development of BC cells, partially due to the inhibition of the PI3K/AKT signaling pathway. **(A)** Following PBS, ZIF-8, ZIF-8@siNC, ZIF-8@siIFI44, ZIF-8@siIFI44@PEG and ZIF-8@siIFI44@PEG-RGD treatment, western blotting was employed to assess the protein expression levels of IFI44 in T24 and J82 cells. **(B)** After being treated with PBS, ZIF-8, ZIF-8@siNC, ZIF-8@siIFI44, ZIF-8@siIFI44@PEG and ZIF-8@siIFI44@PEG-RGD, the viability of BC cells was evaluated. **(C)** BC cell apoptosis was evaluated after PBS, ZIF-8, ZIF-8@siNC, ZIF-8@siIFI44, ZIF-8@siIFI44@PEG and ZIF-8@siIFI44@PEG-RGD treatment. **(D)** The migration capability of BC cells when treated with PBS, ZIF-8, ZIF-8@siNC, ZIF-8@siIFI44, ZIF-8@siIFI44@PEG and ZIF-8@siIFI44@PEG-RGD. **(E)** After co incubating PBS, ZIF-8, ZIF-8@siNC, ZIF-8@siIFI44, ZIF-8@siIFI44@PEG and ZIF-8@siIFI44@PEG-RGD, western blot was used to detect the protein levels of total and phosphorylation of PI3K/AKT. Data were presented as means ± SD. ***P < 0.001; ns, no statistical difference. Experiments were repeated at least three times.

Following the confirmation of ZIF-8@siIFI44@PEG-RGD's biological characteristics, we studied their therapeutic effects on BC cells in vitro. Fig. 4H demonstrated that ZIF-8@siIFI44@PEG-RGD inhibited more than 70% of IFI44 expression at a 40 nM siRNA concentration. Therefore, this dosage was chosen for further experiments. According to western blotting and RT-qPCR assays, the ZIF-8@siIFI44 and ZIF-8@siIFI44@PEG groups led to a reduction in IFI44 expression when compared to the ZIF-8 and ZIF-8@siNC groups. However, the ZIF-8@siIFI44@PEG-RGD group caused a more marked decrease than ZIF-8@siIFI44 and ZIF-8@siIFI44@PEG group (Fig. 5A and Figure S5A, B). Both cell proliferation experiments and cell cloning experiments confirmed that ZIF-8@siIFI44 and ZIF-8@siIFI44@PEG can inhibit the proliferation of BC cells compared with ZIF-8 and ZIF-8@siNC groups, however, ZIF-8@siIFI44@PEG-RGD group has the most significant inhibitory effect (Fig. 5B and Figure S5C, D). The Annexin-V/PI assay also demonstrated that ZIF-8@siIFI44, ZIF-8@siIFI44@PEG groups boosted cell apoptosis activity and ZIF-8@siIFI44@PEG-RGD group exhibited the highest apoptosis efficiency when compared to ZIF-8 and ZIF-8@siNC groups (Fig. 5C and Figure S5E). The Transwell experiment also demonstrated that ZIF-8@siIFI44@PEG-RGD group has the strongest ability to inhibit migration (Fig. 5D and Figure S5F). Overall, ZIF-8@siIFI44 and ZIF-8@siIFI44@PEG showed a notable ability to inhibit the growth of BC cells, while also promoting apoptosis. Additionally, ZIF-8@siIFI44@PEG-RGD amplifies these effects in vitro. To further verify that ZIF-8@siIFI44@PEG-RGD hinders the malignant development of BC cells, partially due to the inhibition of the PI3K/AKT signaling pathway at the level of nanomaterials. BC cells were incubated with various ZIF-8 groups for 48 hours, and changes in total and phosphorylation of the PI3K and AKT were detected using western blot. The results showed that ZIF-8@siIFI44 and ZIF-8@siIFI44@PEG could inhibit the phosphorylation of the PI3K/AKT, while ZIF-8@siIFI44@PEG-RGD had the most significant inhibitory effect. In conclusion, ZIF-8@siIFI44@PEG-RGD hinders the malignant development of BC cells, partially due to the inhibition of the PI3K/AKT signaling pathway.

### In vivo study of the therapeutic potential of ZIF-8@siIFI44@PEG-RGD

**Fig. 6:**
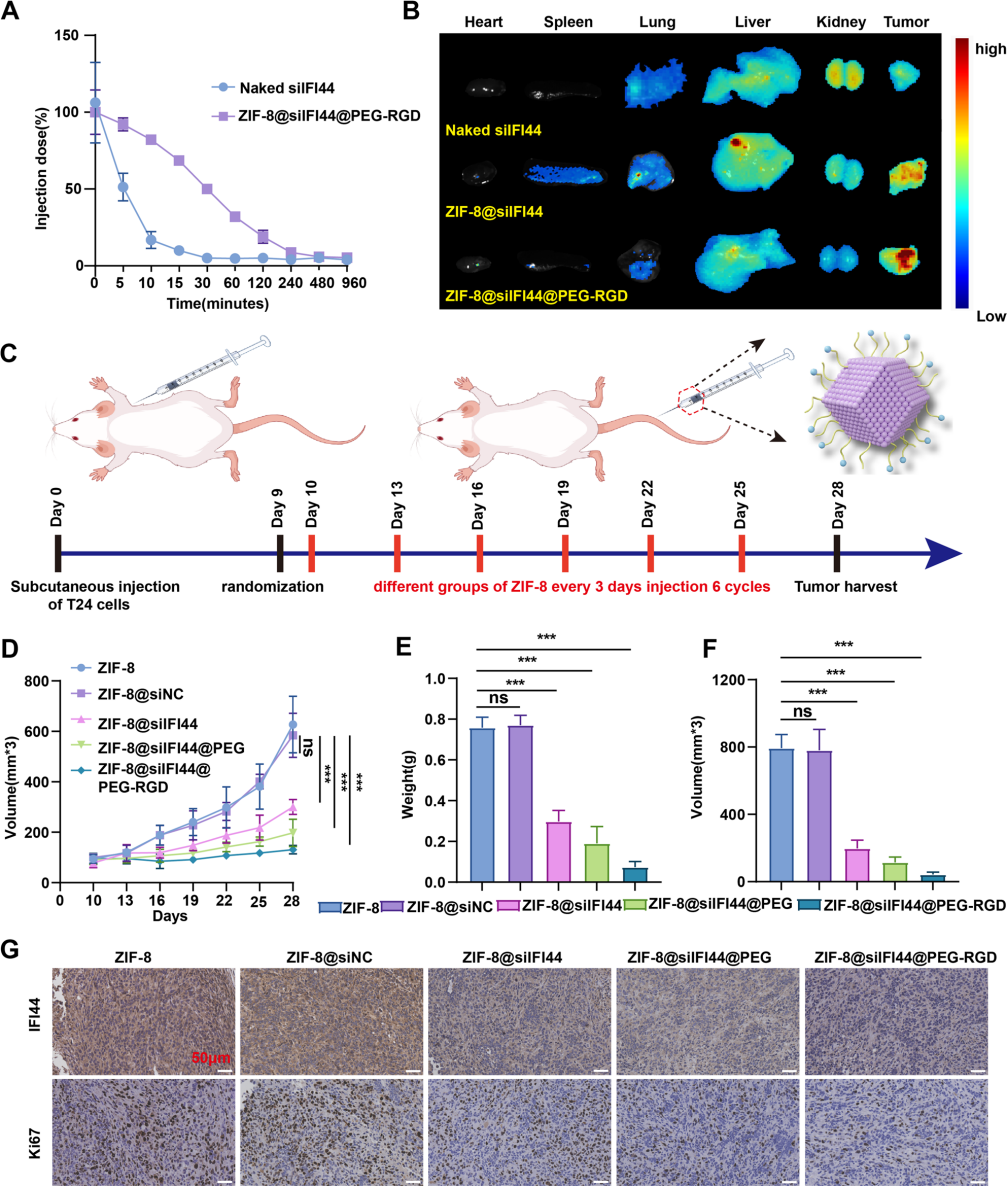
In vivo study of the therapeutic potential of ZIF-8@siIFI44@PEG-RGD. **(A)** In vivo pharmacokinetic evaluations were conducted for both naked siIFI44 and ZIF-8@siIFI44@PEG-RGD. **(B)** After intravenous administration of naked siIFI44, ZIF-8@siIFI44 and ZIF-8@siIFI44@PEG-RGD, major organs and tumors were harvested 24 h later for biodistribution analysis with a small animal CT/live imaging all-in-one device (Milabs B.V.). **(C)** In a schematic diagram, the timeline for implanting tumors of T24 cells and administering ZIF-8, ZIF-8@siNC, ZIF-8@siIFI44, ZIF-8@siIFI44@PEG and ZIF-8@siIFI44@PEG-RGD to mice is illustrated. Various ZIF-8 were injected into the mice every three days, completing six cycles. **(D)** The increase in tumor volume in vivo is illustrated. **(E)** The weight of the detached tumor. **(F)** The volumn of the detached tumor. **(G)** IHC analysis of the expression levels of IFI44 and Ki67 after treatment with ZIF-8, ZIF-8@siNC, ZIF-8@siIFI44, ZIF-8@siIFI44@PEG and ZIF-8@siIFI44@PEG-RGD. Data were presented as means ± SD. ***P < 0.001; ns, no statistical difference. Experiments were repeated at least three times.

Next, we performed a detailed investigation into the pharmacokinetics (PK) and biodistribution (BioD) of ZIF-8@siIFI44@PEG-RGD in vivo. We used healthy nude mice to perform pharmacokinetics. A random allocation process was used to divide healthy nude mice into two groups. Naked siIFI44 and ZIF-8@siIFI44@PEG-RGD were injected intravenously into healthy mice at a 1 nmol siRNA dose (*n* = 3). Fig.  6A showed that naked siIFI44 was quickly removed from the bloodstream, while ZIF-8@siIFI44@PEG-RGD had extended half-lives, with about 20% of the initial dose still present two hours after injection. Following this, we used a mouse xenograft tumor model to conduct biodistribution (BioD). All nude mice were administered subcutaneous injections of approximately 5 million T24 cells. Once the tumor volume reached 100 mm³, the nude mice receiving an intravenous injection of either naked siIFI44, ZIF-8@siIFI44 or ZIF-8@siIFI44@PEG-RGD. Afte tail vein injection of naked siIFI44, ZIF-8@siIFI44 and ZIF-8@siIFI44@PEG-RGD 24 h, tumors and major organs were collected for biological distribution analysis. Naked siIFI44 is mostly found in the kidneys, then in the liver, with almost no fluorescence in tumor tissues. On the other hand, ZIF-8@siIFI44@PEG-RGD demonstrated significantly higher fluorescence accumulation in tumor compared to naked siIFI44 and ZIF-8@siIFI44 (Fig. 6B**)**. The outcome further verified that ZIF-8@siIFI44@PEG-RGD is capable of specifically targeting tumor tissue. Therefore, PK and BioD experiments have demonstrated the effective retention of ZIF-8@siIFI44@PEG-RGD in the blood and its efficient targeting to tumor tissues. To evaluate the anti-tumor effectiveness of ZIF-8@siIFI44@PEG-RGD, a xenograft tumor model was created by injecting T24 cells into the mice’s axillary area. All nude mice (*n* = 25) received subcutaneous injections of approximately 5 million T24 cells. On the 9th day, the tumor-bearing mice were randomly allocated into five groups (*n* = 5). Commencing on the 10th day, the mice were administered injections of ZIF-8, ZIF-8@siNC, ZIF-8@siIFI44, ZIF-8@siIFI44@PEG and ZIF-8@siIFI44@PEG-RGD every three days, totaling six consecutive injections, until euthanasia on the 28th day (Fig. 6C). Compared to the ZIF-8, and ZIF-8@siNC groups, those mice treated with ZIF-8@siIFI44 and ZIF-8@siIFI44@PEG showed a decrease in tumor volume. In particular, the ZIF-8@siIFI44@PEG-RGD group had the smallest tumor volume (Fig. 6D), suggesting that ZIF-8@siIFI44 and ZIF-8@siIFI44@PEG have anti-cancer capabilities, and using with RGD enhances its effect. The tumor weight and volume of the dissected tumor (Fig. 6E, F), all indicated that ZIF-8@siIFI44 and ZIF-8@siIFI44@PEG have tumor-inhibiting properties, which are enhanced after merging with RGD. The protein levels of IFI44 in groups of ZIF-8@siIFI44, ZIF-8@siIFI44@PEG and ZIF-8@siIFI44@PEG-RGD were significantly diminished compared to the other two groups, as confirmed by immunohistochemical analysis, which indicated a successful knockdown of IFI44 in vivo. Moreover, Ki67 assays confirmed that ZIF-8@siIFI44 and ZIF-8@siIFI44@PEG had anti-tumor effects, and when used with RGD, they showed a synergistic anti-tumor benefit (Fig. 6G and Figure S6A, B). Ultimately, ZIF-8@siIFI44@PEG-RGD displayed considerable anti-tumor characteristics in vivo, offering strong evidence for possible clinical use.

### In vivo study of the toxicity of ZIF-8@siIFI44@PEG-RGD

**Fig. 7:**
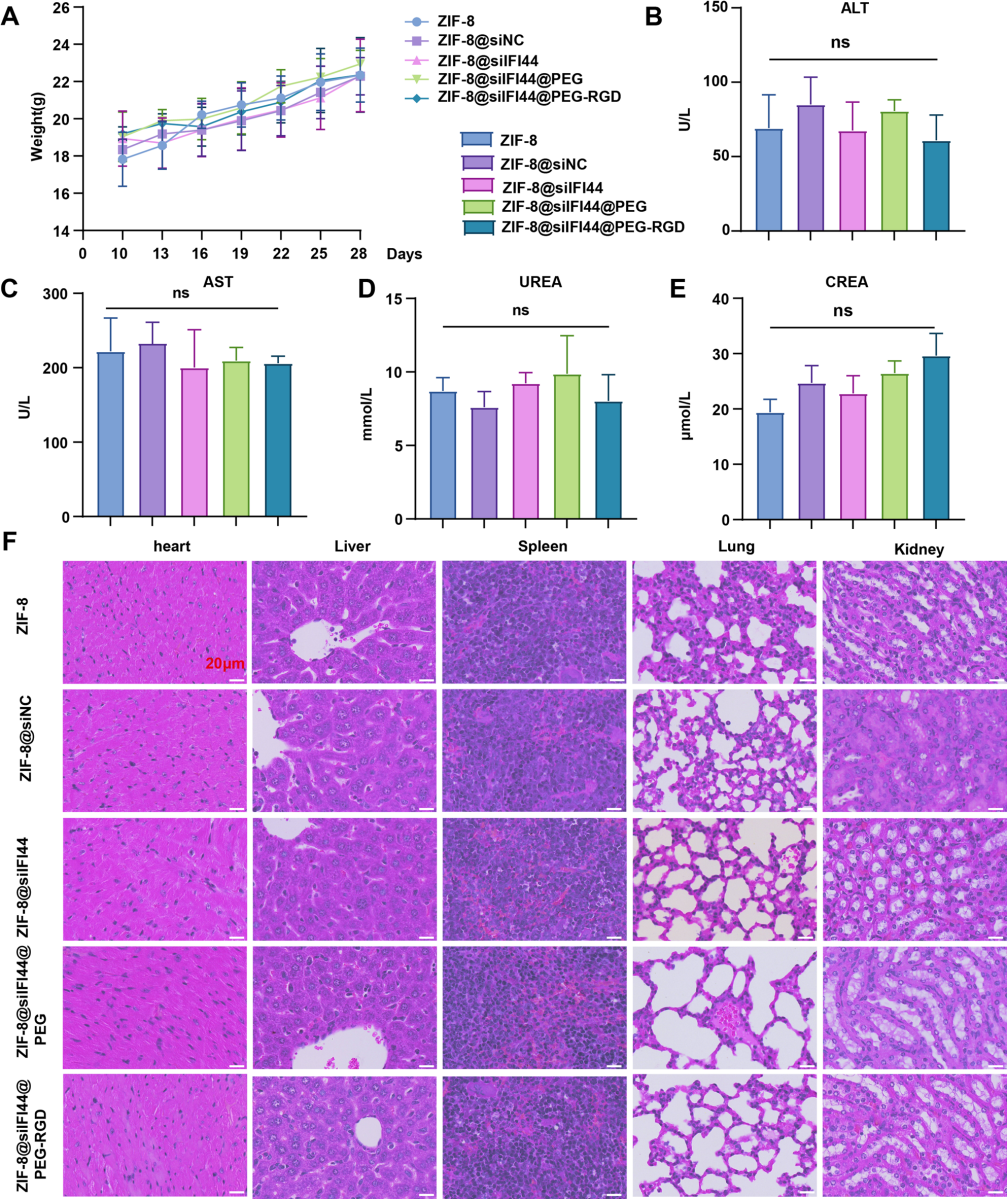
In vivo study of the toxicity of ZIF-8@siIFI44@PEG-RGD. **(A)** Nude mice weight in the various group. **(B**,** C**,** D**,** E)** The measurement of serum ALT, AST, CREA, UREA levels were conducted after administering ZIF-8, ZIF-8@siNC, ZIF-8@siIFI44, ZIF-8@siIFI44 and ZIF-8@siIFI44@PEG-RGD. **(F)** After treatment with ZIF-8, ZIF-8@siNC, ZIF-8@siIFI44, ZIF-8@siIFI44@PEG and ZIF-8@siIFI44@PEG-RGD, images of important organs stained with hematoxylin and eosin (H&E) are displayed, with scale bar: 20 μm. Data were presented as means ± SD. ns, no statistical difference. Experiments were repeated at least three times.

The widespread application of nanomaterials is mainly determined by their potential negative effects on humans. In vivo toxicity experiments were performed on tumor-bearing mice assigned to different ZIF-8 treatment groups, as illustrated in Fig. 6C. Commencing on the 10th day, the body weight of the mice was recorded, and assessments were conducted every three days until the 28th day. Prior to euthanizing the mice on the 28th day, blood samples were obtained from the orbital sinus to conduct biochemical assays for evaluating liver and kidney function. Following euthanasia, in addition to tumor excision, the heart, liver, spleen, lung, and kidney of each mouse were collected for hematoxylin and eosin (HE) staining. These experimental procedures were implemented to assess the in vivo safety profile of ZIF-8. No substantial differences in body weight were observed among the mouse groups after treatment, indicating the treatment’s safety (Fig. 7A). Next, we tested the liver and kidney indicators such as ALT, AST, UREA, CREA in the blood, The standard reference range for serum biochemical markers are as follows: ALT (10.06–96.47) U/L, AST (36.31-235.48) U/L, UREA (3.9–12.4) mmol/L and CREA (10.91–85.09)µmol/L. The results showed that there was no difference between the ZIF-8@siIFI44@PEG-RGD groups and the other four groups (Fig. 7B, C, D, E), confirming the safety of ZIF-8 treatment. Ultimately, we employed H&E staining to assess systemic toxicity, and the findings indicated that mice treated with various ZIF-8 did not exhibit significant damage to the heart, liver, spleen, lung, and kidney (Fig. 7F). This outcome further confirms the safety of ZIF-8 treatment. Overall, ZIF-8@siIFI44@PEG-RGD did not lead to notable toxicity in the body’s primary organs, offering substantial proof for possible clinical use.

## Discussion

Excessive activation of abnormal signaling pathways can lead to malignant progression in BC, hindering effective treatment. BC’s progression is impeded by the knockdown of IFI44, partially due to the inhibition of the PI3K/AKT signaling pathway. We engineered a ZIF-8 nanoplatform for targeted delivery of siIFI44 to BC tissues, facilitating the clinical application of this discovery. Our research indicated that ZIF-8 loaded with siIFI44 exhibits anti-tumor effects on BC. The ZIF-8@siIFI44@PEG-RGD formulation showed notable anti-tumor effectiveness, offering a fresh outlook for treatment strategies against the disease.

The involvement of IFI44 in tumorigenesis has been documented in the literature, with evidence suggesting its dual role in either promoting or inhibiting tumor development. Its potential utility in cancer prognosis has been highlighted through various studies [44, 45]. LINC01116 influences IFI44 expression, impacting gefitinib resistance, indicating IFI44 as a potential target to counteract drug resistance in non-small cell lung cancer [15]. Research has demonstrated that bioactive dietary components have the potential to influence tumor proliferation and development through the regulation of gene expression, including that of IFI44 [46]. In summary, the expression of IFI44 is significantly linked to tumor development, progression, and prognosis across various cancer types. However, its specific role in BC remains unproven. This article aims to elaborate the IFI44's role in BC and explore its clinical applications.

To elaborate the IFI44's mechanisms in BC cell, we used RNA sequencing to identify alterations in associated signaling pathways following the knockdown of IFI44. The results demonstrated a notable enrichment of the PI3K/AKT signaling pathway. Protein level validation proved that the knockdown of IFI44 inhibited BC malignant progression partially by suppressing the phosphorylation of the PI3K/AKT pathway. We then used 740 Y-P, a cell-permeable and potent PI3K activator, for supplementation experiments. In vitro and in vivo experiments confirmed the inactivity of the PI3K/AKT signing pathway after the knockdown of IFI44. Although PI3K/AKT/mTOR inhibitors have demonstrated potential in certain malignancies, their effectiveness as standalone treatments is restricted [47]. In triple-negative breast cancer (TNBC), the limited efficacy of PI3K/AKT/mTOR inhibitors might be attributed to persistent RB protein phosphorylation and inadequate p-S633 suppression [48]. The clinical use of these inhibitors often leads to notable toxicity and drug resistance, reducing their therapeutic effectiveness [49]. Future research endeavors could prioritize the optimization of inhibitor specificity, the identification of suitable biomarkers, and the investigation of combination therapies to augment the clinical efficacy of PI3K/AKT pathway inhibitors [50]. This study proposes that combining the knockdown of IFI44 with PI3K inhibition could improve therapeutic outcomes for BC.

At present, there is a notable paucity of authoritative literature addressing the association between IFI44 and BC. Our research has uniquely identified and validated the correlation. Our findings suggest that IFI44 can partially regulate the phosphorylation of the PI3K/AKT pathway. However, this study has not yet explored the specific underlying mechanisms, such as the particular genes through which IFI44 influences the phosphorylation of the PI3K/AKT pathway. Further investigation is needed to explore the connection between IFI44 and the PI3K/AKT pathway. We intend to employ mass spectrometry analysis to identify genes that interact with IFI44. Subsequently, co-immunoprecipitation (Co-IP) experiments, proximity ligation assays (PLA), and immunofluorescence (IF) experiments will be conducted to validate potential candidate genes. Subsequent research will delve into how IFI44 regulates interacting genes through processes such as ubiquitination, phosphorylation, or methylation.

The targeted therapeutic application of ZIF-8 is primarily facilitated by its exceptional drug delivery capabilities and multifunctional properties. ZIF-8 has been widely studied for its potential in targeted tumor therapy. The fusion of ZIF-8 with various functional materials facilitates diverse anti-tumor treatment approaches [51, 52]. Additionally, ZIF-8 exhibits considerable potential in immunotherapy applications [53, 54]. ZIF-8 nanomaterials have recently attracted significant interest in gene-targeted therapy. ZIF-8, a MOF material, is considered an ideal gene delivery carrier due to its excellent biocompatibility and pH-responsive properties. Studies have demonstrated that ZIF-8 can effectively protect nucleic acids from enzymatic degradation and release the encapsulated gene drugs in acidic environments, thereby enabling targeted therapy [55, 56]. In oncological therapeutics, ZIF-8 has been widely employed in gene delivery systems. A notable example is the Il2/ZIF-8@Salmonella system, wherein ZIF-8 conjugates with Salmonella to facilitate targeted gene delivery within the tumor microenvironment. This system markedly enhances anti-tumor efficacy by activating cytotoxic T cells and M1-type macrophages [57]. Moreover, ZIF-8 has been employed for small interfering RNA (siRNA) delivery. In prostate cancer treatment, ZIF-8-mediated delivery of SNHG15 siRNA effectively suppresses tumor cell growth and triggers apoptosis [56]. The versatility of ZIF-8 extends beyond gene delivery, encompassing applications in combination therapies. When integrated with polydopamine nanoparticles, ZIF-8 facilitates tumor-specific accumulation of siRNA and significantly enhances therapeutic efficacy, as demonstrated through photoacoustic/near-infrared dual-modal imaging guidance [58]. Furthermore, ZIF-8 functions as an adjuvant in chemotherapeutic kinetics by synergizing with miRNA delivery to enhance tumor cell apoptosis [59]. While the application status and future development trajectory of the nanomaterial ZIF-8 in BC treatment have emerged as prominent research focal points, its clinical application continues to encounter challenges. Future research endeavors should prioritize the optimization of ZIF-8’s structure and function to improve its stability and biocompatibility in vivo. Exploring the combination of ZIF-8 with other therapeutic approaches for enhanced BC treatment offers a promising research direction [60, 61]. Through ongoing technological advancements and clinical trials, ZIF-8 is anticipated to assume a more significant role in the diagnosis and treatment of BC. This study engineered ZIF-8, conjugated with an RGD-targeted peptide, as a delivery vehicle for siIFI44 to specifically transport it to BC cells. This design enables the entry of the complex into the cytoplasm, thereby achieving the silencing of IFI44.

## Conclusions

In our study, we observed a significant upregulation of IFI44 expression in BC cells and tissues. Mechanistically, BC’s progression is impeded by the knockdown of IFI44, partially due to the inhibition of the PI3K/AKT signaling pathway. Notably, our research team has engineered a ZIF-8 nanoplatform capable of effectively encapsulating siIFI44, which has demonstrated anti-tumor efficacy through inhibition of the phosphorylation of the PI3K/AKT, as illustrated in the. Consequently, targeting the IFI44-PI3K/AKT axis may offer a novel therapeutic strategy for BC.

## Supplementary Information

Supplementary Material 1

Supplementary Material 2

## Data Availability

The data utilized in this research can be accessed from Aiping Le (ndyfy00973@ncu.edu.cn) upon a reasonable request.
