# Supplementary material for: The ZIF-8 nanoplatform targeted delivery of IFI44 siRNA to suppress bladder cancer development via modulating the PI3K/AKT signaling pathway
Source: J Nanobiotechnology. 2026 Apr 30;24:580. doi: 10.1186/s12951-026-04419-w (PMC13285535; doi:10.1186/s12951-026-04419-w)

Full unedited gel for Figure 1E

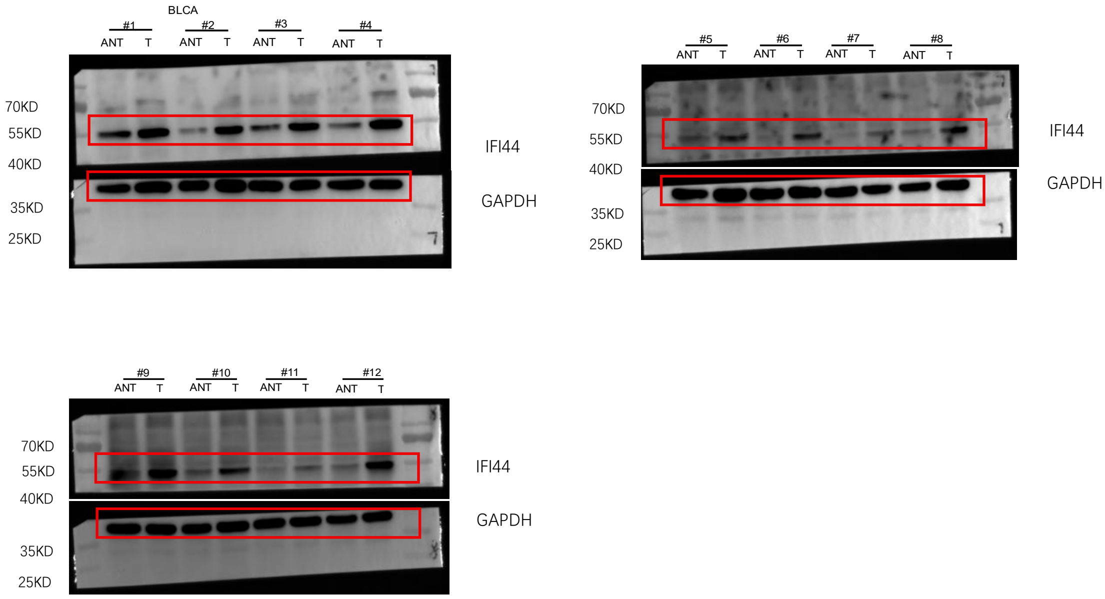

Full unedited gel for Figure 1G

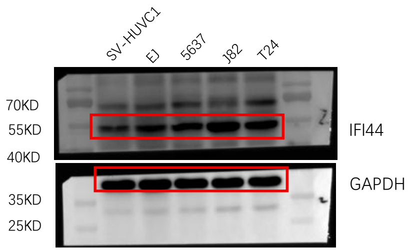

Full unedited gel for Figure 2B

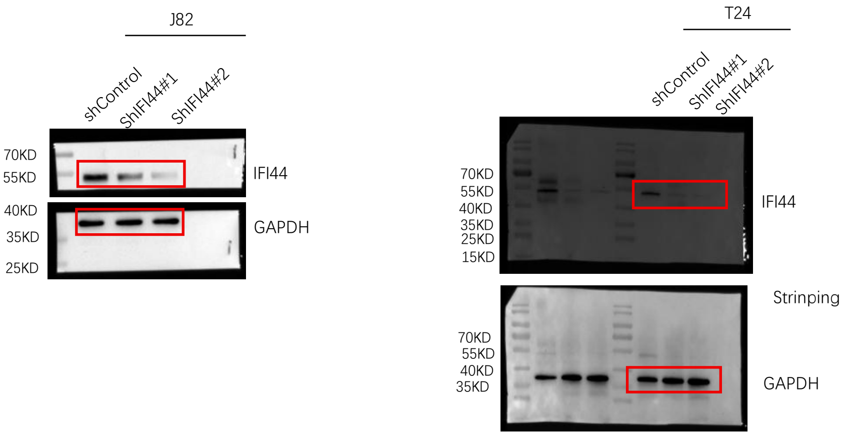

Full unedited gel for Figure 2E

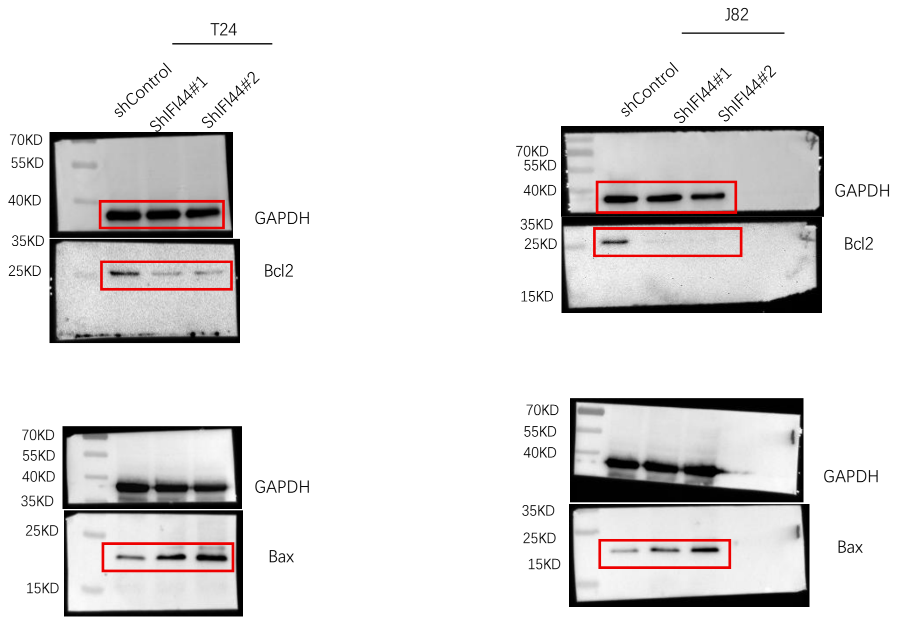

Full unedited gel for Figure 3B

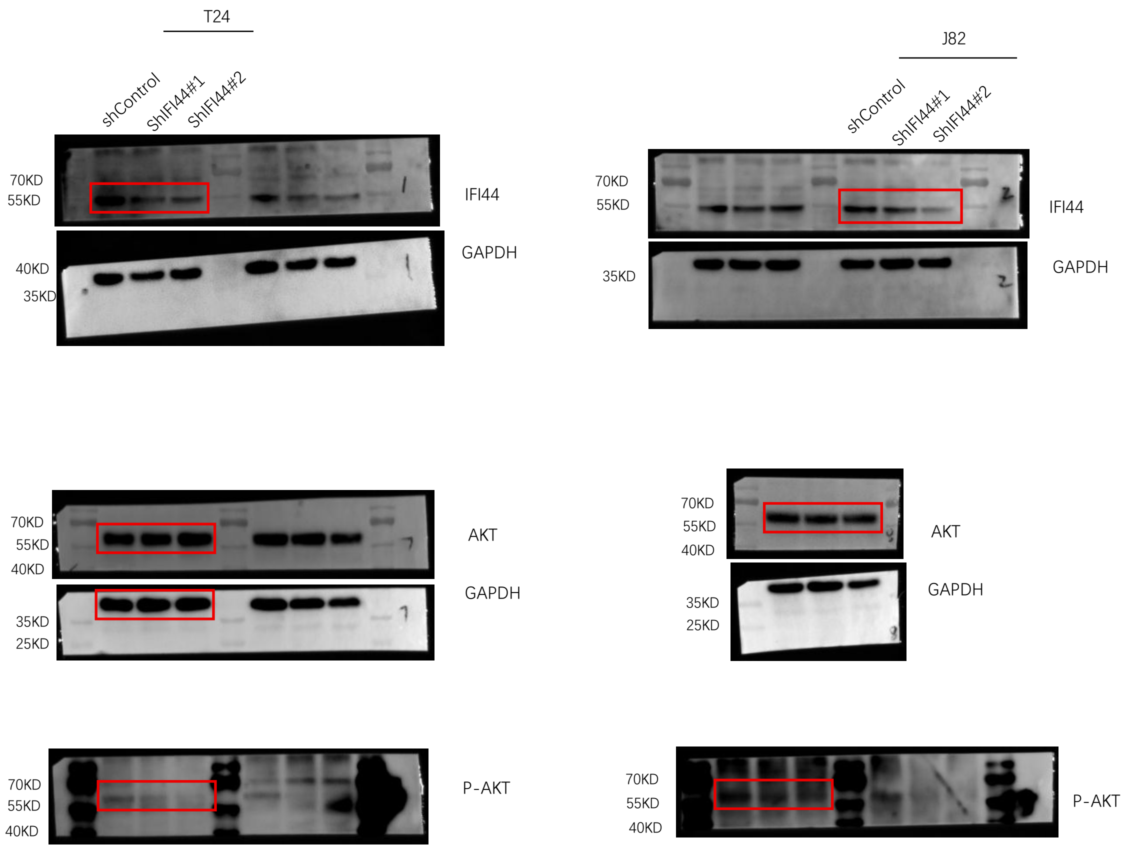

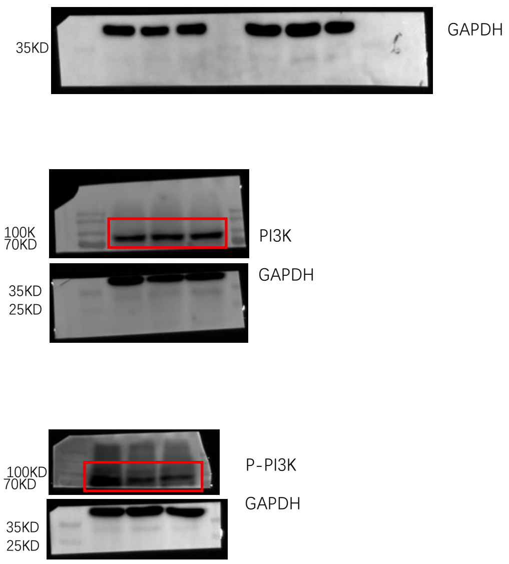

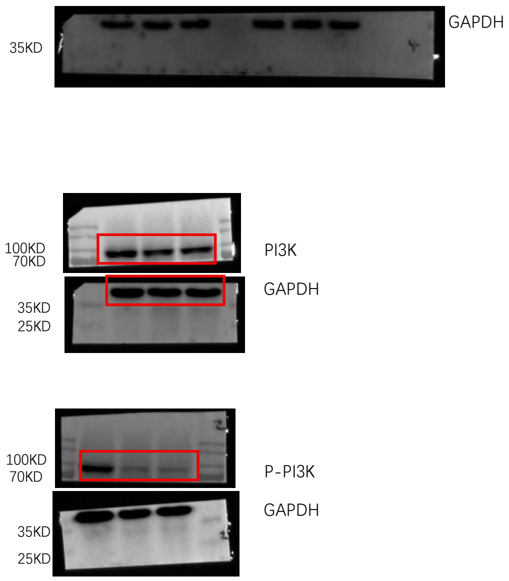

Full unedited gel for Figure 3C

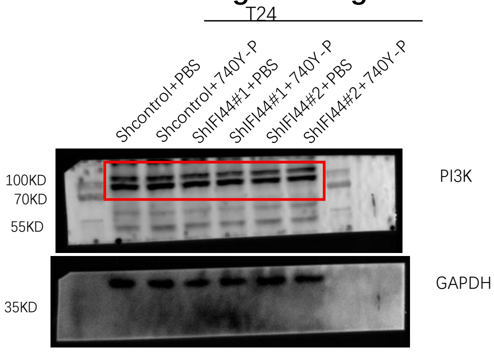

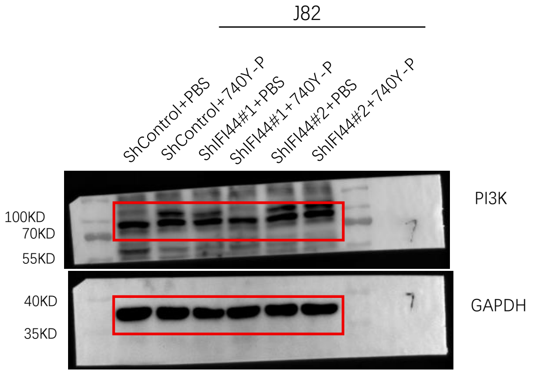

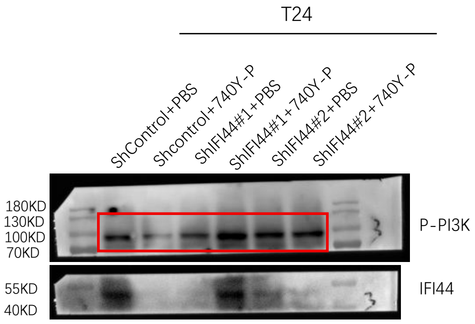

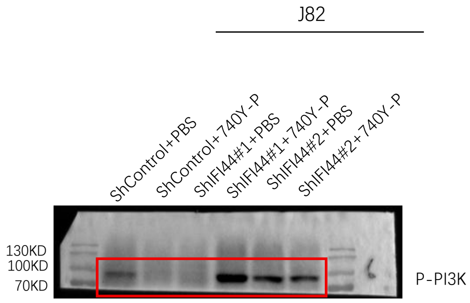

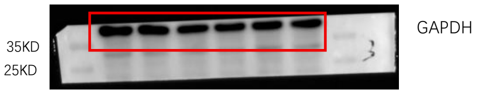

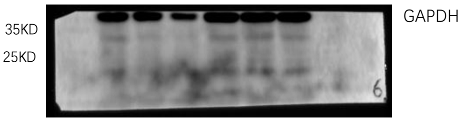

Full unedited gel for Figure 4H

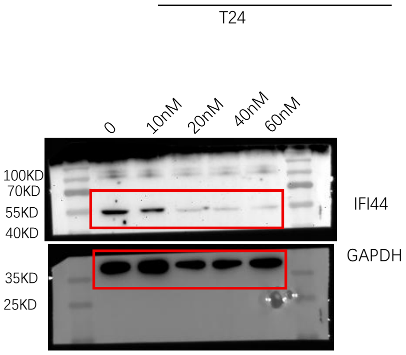

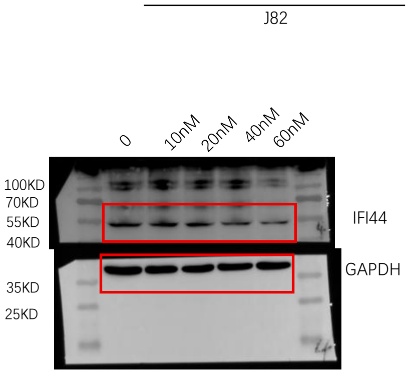

Full unedited gel for Figure 5A

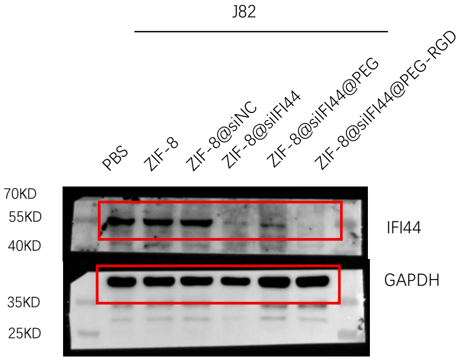

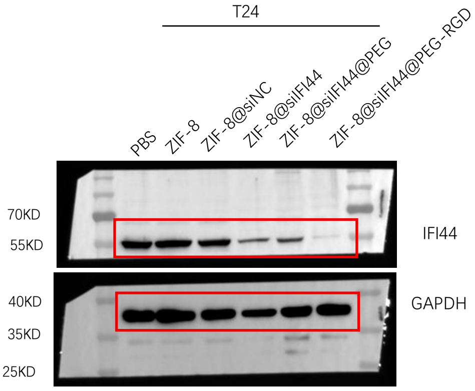

Full unedited gel for Figure 5E

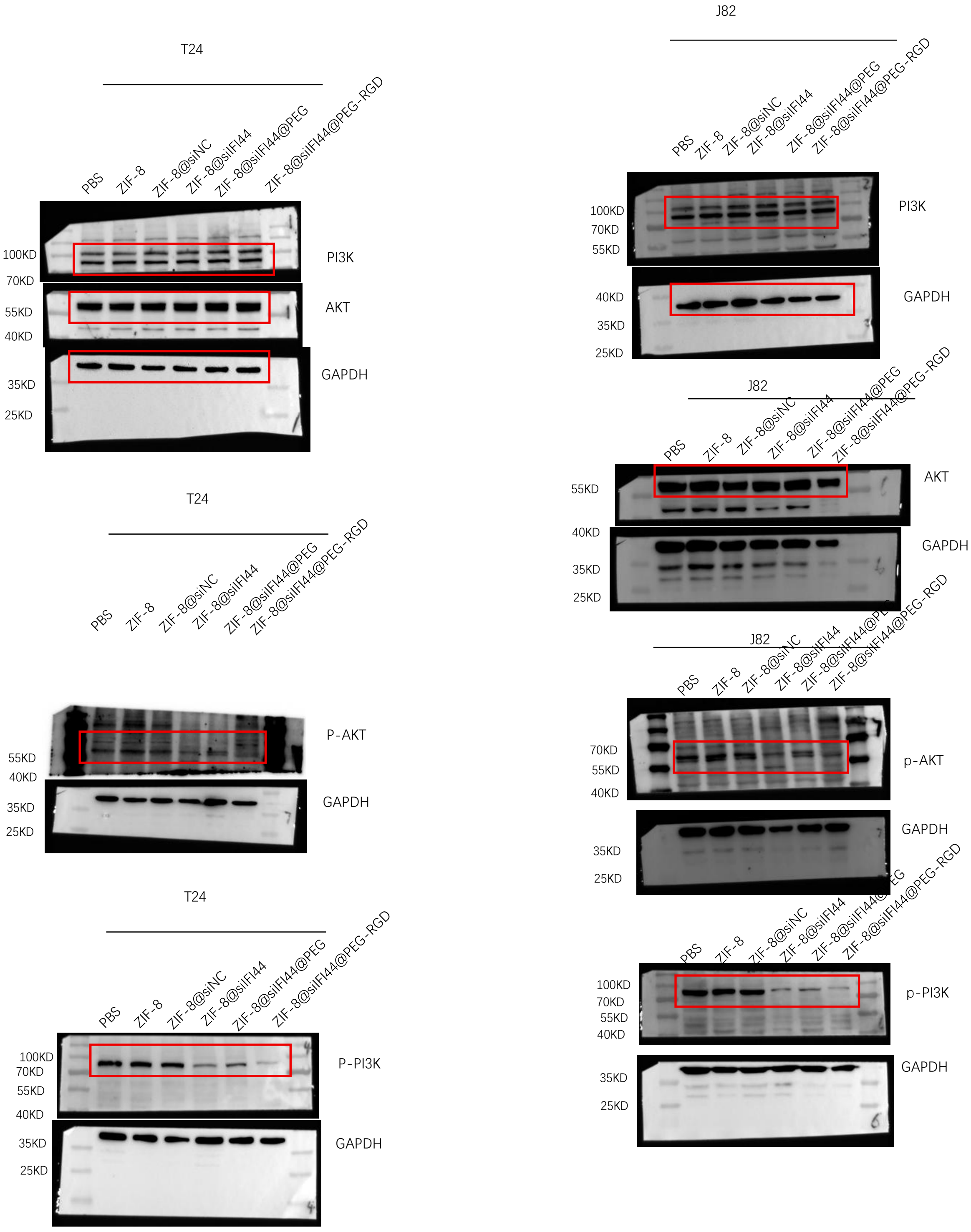

Supplement: Supplementary file 1 — Supplementary Material 1 [file 12951_2026_4419_MOESM1_ESM.pdf]
