# Supplementary material for: The ZIF-8 nanoplatform targeted delivery of IFI44 siRNA to suppress bladder cancer development via modulating the PI3K/AKT signaling pathway
Source: J Nanobiotechnology. 2026 Apr 30;24:580. doi: 10.1186/s12951-026-04419-w (PMC13285535; doi:10.1186/s12951-026-04419-w)
Supplement: Supplementary file 2 — Supplementary Material 2 [file 12951_2026_4419_MOESM2_ESM.docx]

Supplementary Materials for

**The ZIF-8 nanoplatform targeted delivery of IFI44 siRNA to suppress bladder cancer development via modulating the PI3K/AKT signaling pathway.**

Chao Zhu^1^**^†^**, Mengwei Liu^2^**^†^**, Xiaohua Liu^3,4^**^†^**, Sifan Zhang^1^, Yun He^1^, Meng Chen^5^, Haoxuan Huang^2^*, Kuai Yu^1^*, Aiping Le^1^*

Correspondence to: [ndyfy00973@ncu.edu.cn](mailto:ndyfy00973@ncu.edu.cn)

**This PDF file includes:**

Figures S2 to S6

Tables. S1

**
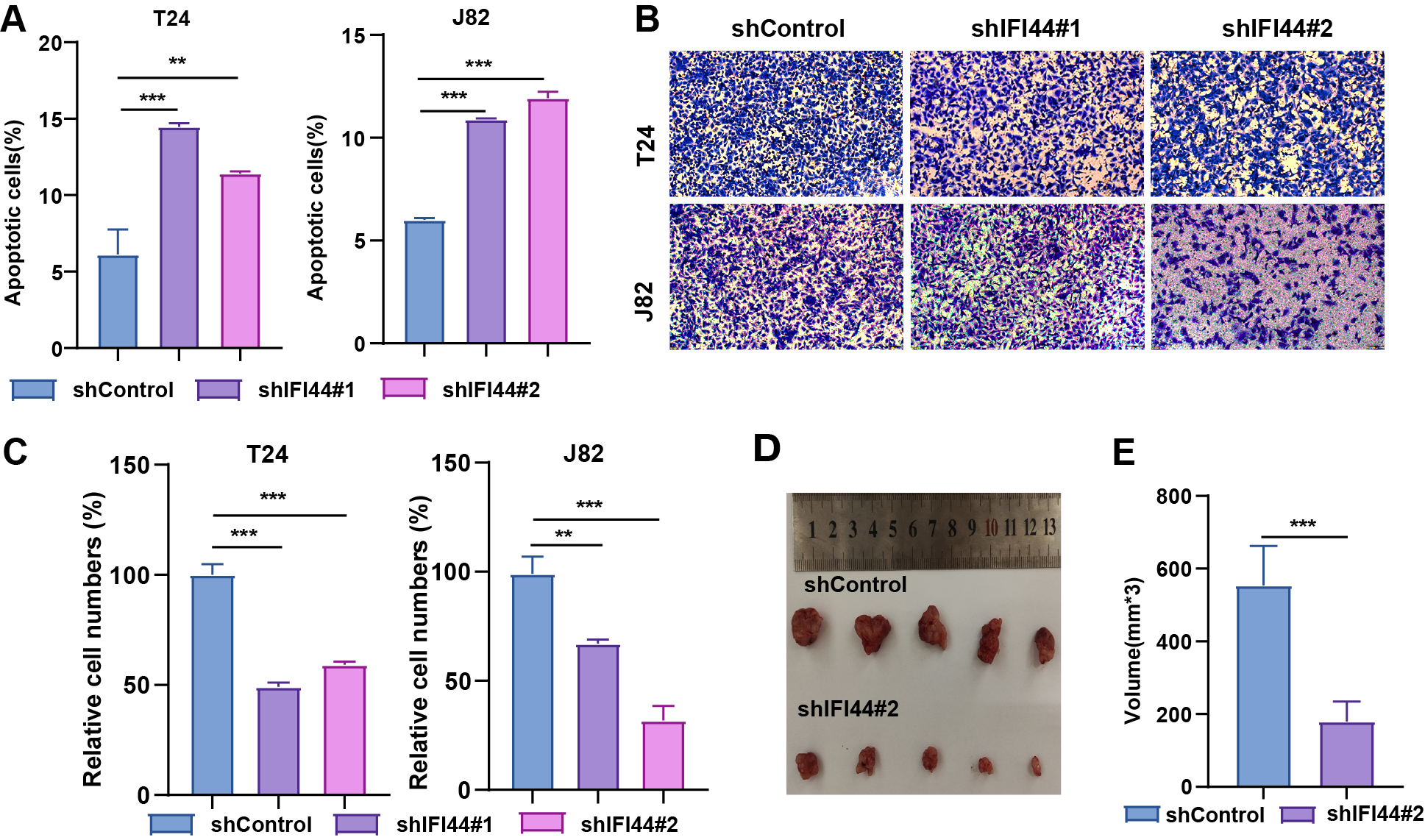
**

**Figure S2 (A)** Cell apoptosis rate of BC cells with the knockdown of IFI44. **(B)** Cell migration ability of BC cells with the knockdown of IFI44. **(C)** Cell migration rate of BC cells with the knockdown of IFI44. **(D)** Visual depiction of tumor samples. **(E)** Measurement of tumor volume post-excision. Data are expressed as means ± standard deviation (SD). ***P*<0.01; ****P*<0.001. Experiments were repeated at least three times.

**
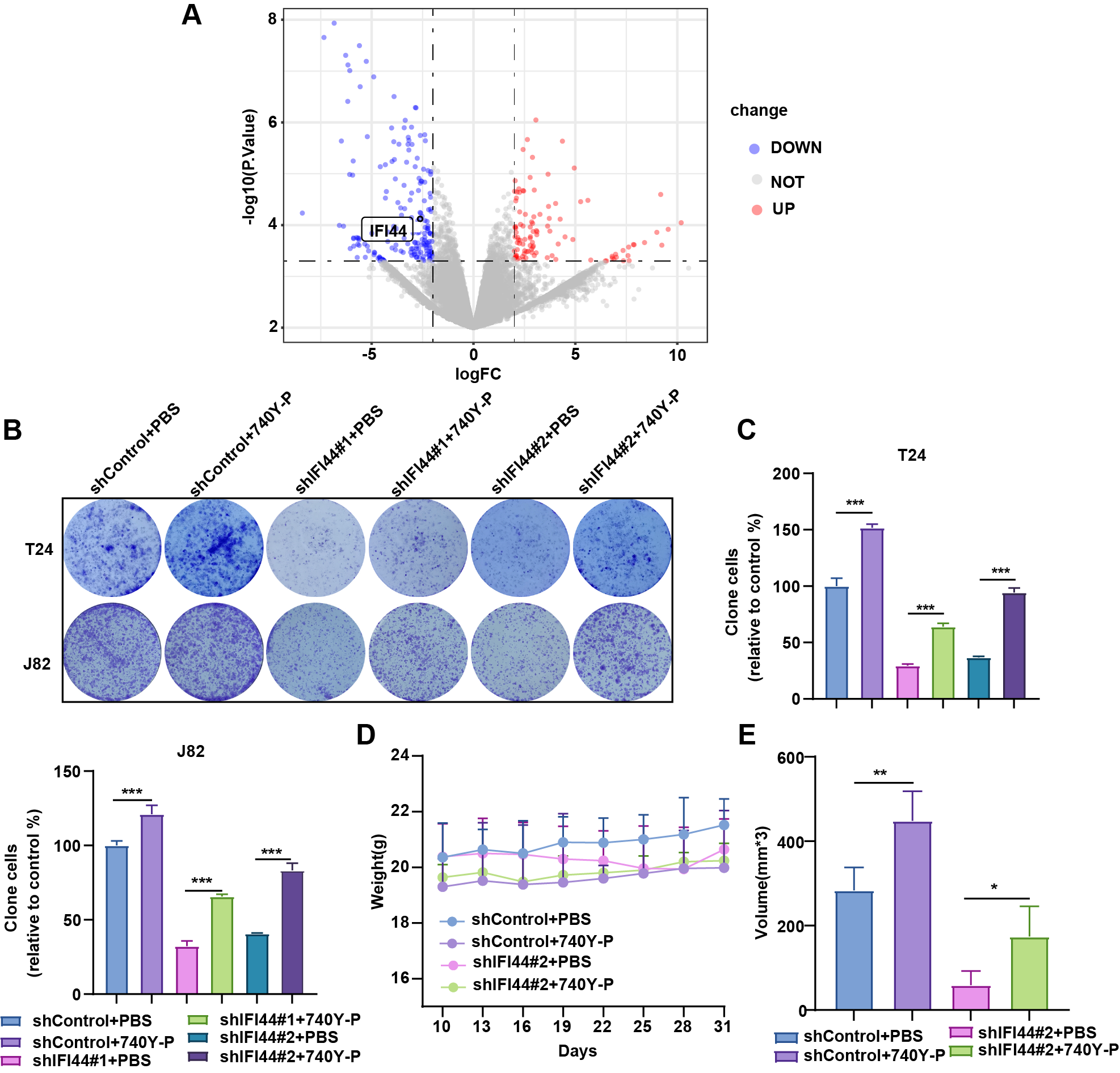
**

**Figure S3 (A)** Volcanic map after knocking down of IFI44. **(B-C)** Cell clone assay was employed to assess the proliferative impact of 740Y-P on BC cells with IFI44 knockdown. **(D)** Nude mice weight in the various groups. **(E)** Measurement of tumor volume post-excision. Data are expressed as means ± standard deviation (SD). **P*<0.05; ***P*<0.01; ****P*<0.001. Experiments were repeated at least three times.


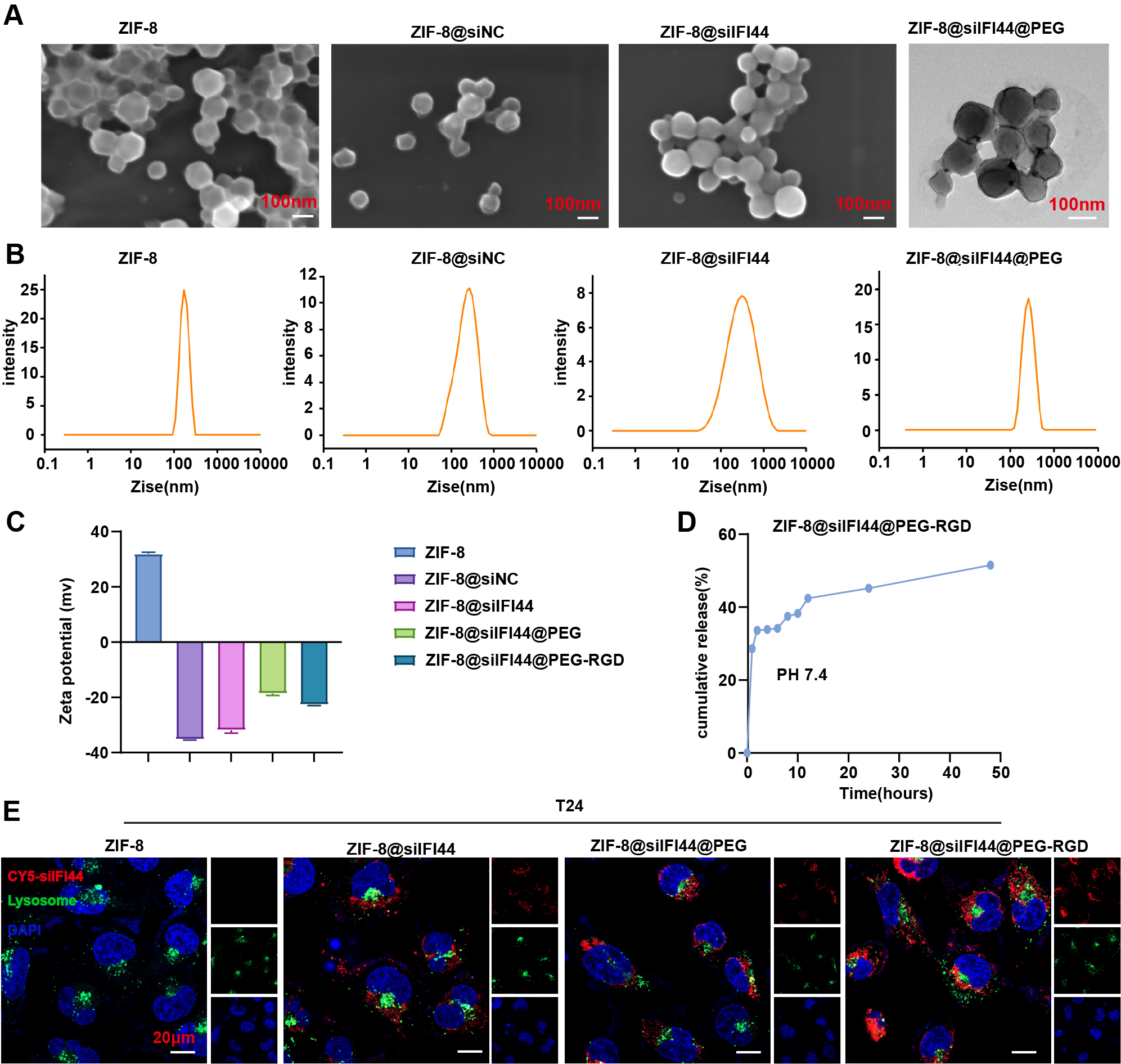


**Figure S4 (A-C)** Scanning electron microscope images of ZIF-8, ZIF-8@siNC, ZIF-8@siIFI44 and transmission electron microscopy image of ZIF-8@siIFI44@PEG, with scale bars: 100 nm. **(B)** Dynamic light scattering analysis was employed to determine the particle sizes of ZIF-8, ZIF-8@siNC, ZIF-8@siIFI44 and ZIF-8@siIFI44@PEG. **(C)** Dynamic light scattering analysis was employed to determine the zeta potentials of ZIF-8, ZIF-8@siNC, ZIF-8@siIFI44 and ZIF-8@siIFI44@PEG. **(D)** Cumulative release profile of siIFI44 in pH7.4 from ZIF-8@siIFI44@PEG-RGD at various time intervals. **(E)** Confocal laser scanning microscopy images of T24 cells following a 4-hour incubation with ZIF-8, ZIF-8@siIFI44, ZIF-8@siIFI44@PEG and ZIF-8@siIFI44@PEG-RGD. Nuclei were stained blue using Hoechst 33342, endosomes were visualized in green with Lysotracker and siIFI44 was labeled with CY5, with scale bars: 20μm. Experiments were repeated at least three times.


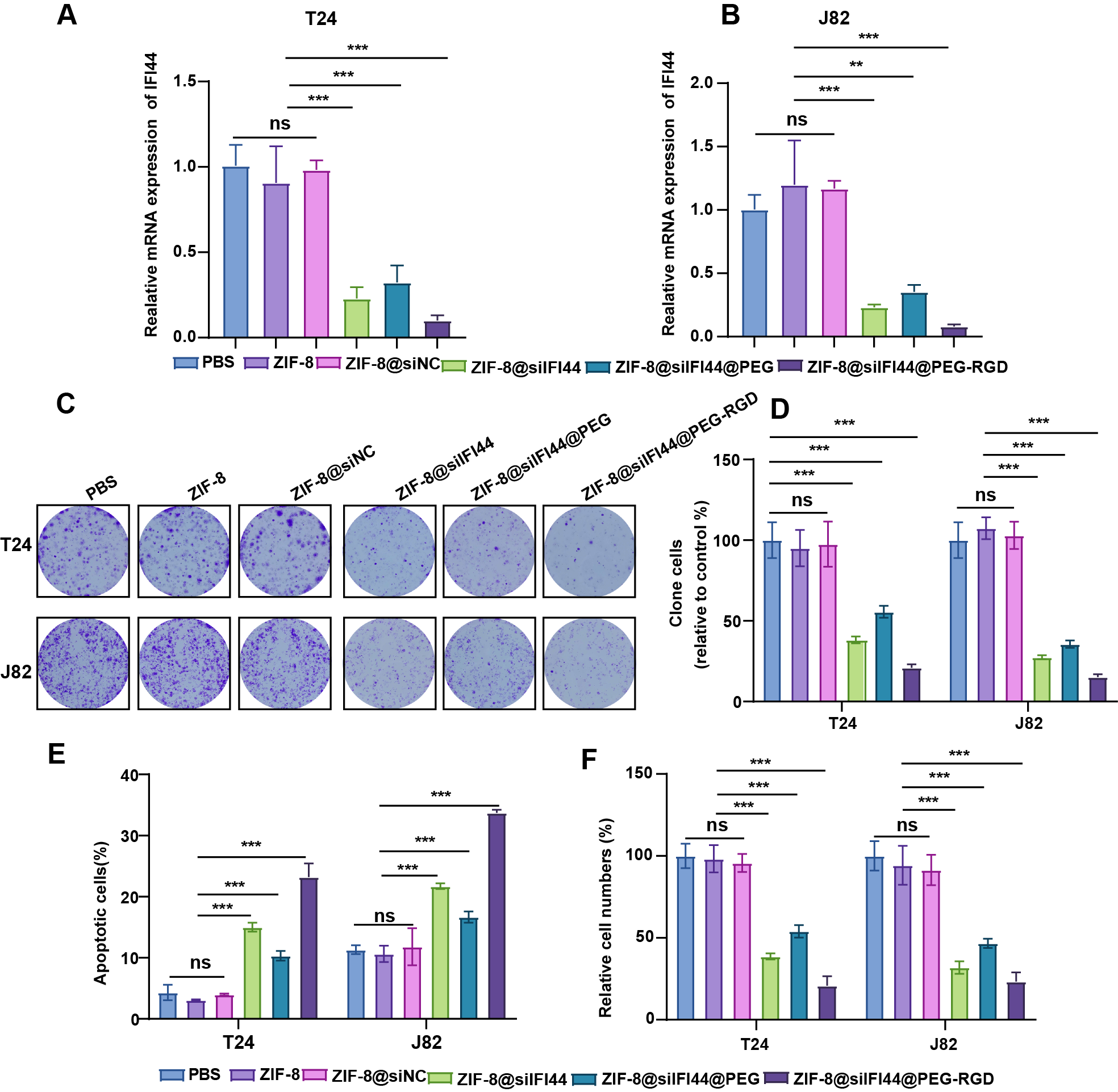


**Figure S5 (A-B)** Following PBS, ZIF-8, ZIF-8@siNC, ZIF-8@siIFI44, ZIF-8@siIFI44@PEG and ZIF-8@siIFI44@PEG-RGD treatment, RT-qPCR was employed to assess the RNA expression levels of IFI44 in T24 and J82 cells. **(C-D)** Cell clone experiment was evaluated after PBS, ZIF-8, ZIF-8@siNC, ZIF-8@siIFI44, ZIF-8@siIFI44@PEG and ZIF-8@siIFI44@PEG-RGD treatment. **(E)** Cell apoptosis rate was evaluated after PBS, ZIF-8, ZIF-8@siNC, ZIF-8@siIFI44, ZIF-8@siIFI44@PEG and ZIF-8@siIFI44@PEG-RGD treatment. **(F)** Cell migration rate was evaluated after PBS, ZIF-8, ZIF-8@siNC, ZIF-8@siIFI44, ZIF-8@siIFI44@PEG and ZIF-8@siIFI44@PEG-RGD treatment. Data are expressed as means ± standard deviation (SD). ***P*<0.01; ****P*<0.001; ns, no statistical difference. Experiments were repeated at least three times.


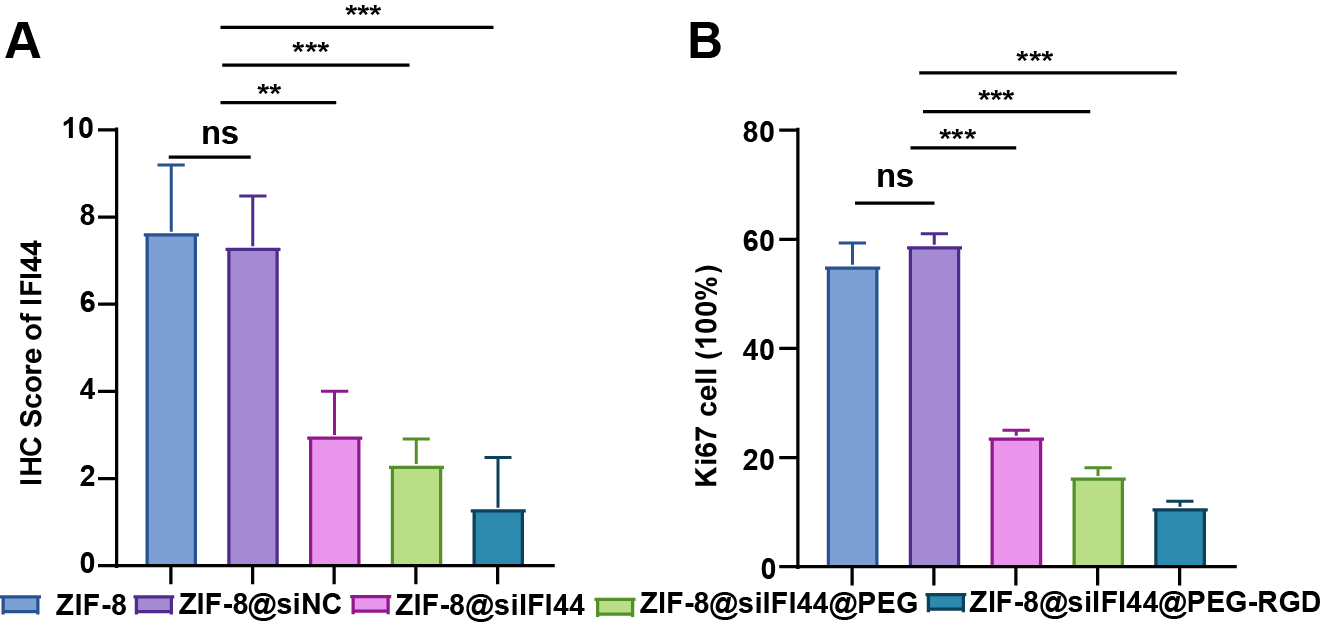


**Figure S6 (A-B)** IHC score of the expression levels of IFI44 and Ki67 after treatment with ZIF-8, ZIF-8@siNC, ZIF-8@siIFI44, ZIF-8@siIFI44@PEG and ZIF-8@siIFI44@PEG-RGD. Data were presented as means±SD. ***P*<0.01; ****P*<0.001; ns, no statistical difference. Experiments were repeated at least three times.

**Table S1 Primer Sequences for RT-qPCR, siRNA and shRNA**

1

| β-actin | F | 5′-CACCATTGGCAATGAGCGGTTC-3′ | RT-qPCR |
| --- | --- | --- | --- |
| IFI44 | R  F  R | 5′-AGGTCTTTGCGGATGTCCACGT-3′  5′-GTGAGGTCTGTTTTCCAAGGGC-3′  5′-CGGCAGGTATTTGCCATCTTTCC-3′ |  |

2

| siNC  siIFI44 | Sense  Anti-sense  Sense  Anti-sense | 5’-UUCUCCGAACGUGUCACGUTT-3′  5’-ACGUGACACGUUCGGAGAATT-3′  5-AGGAUAACCUAGACGACAUAATT-3′  5-UUAUGUCGUCUAGGUUAUCCUTT-3′ | siRNA |
| --- | --- | --- | --- |

3

| ShIFI44#1  ShIFI44#2 | Sense  Anti-sense  Sense  Anti-sense | 5’-GATCGAGGATAACCTAGACGACATAACTCGAGTTATGTCGTCTAGGTTATCCTTTTTTG-3′  5’-AATTCAAAAAAGGATAACCTAGACGACATAACTCGAGTTATGTCGTCTAGGTTATCCTC -3′  5’-GATCGACCGAGCGGTATAGGATATATCTCGAGATATATCCTATACCGCTCGGTTTTTTG -3′  5’-AATTCAAAAAACCGAGCGGTATAGGATATATCTCGAGATATATCCTATACCGCTCGGTC -3′ | shRNA |
| --- | --- | --- | --- |
